# Evaluation of the immunomodulatory effects of interleukin-10 on peripheral blood immune cells of COVID-19 patients: Implication for COVID-19 therapy

**DOI:** 10.3389/fimmu.2022.984098

**Published:** 2022-09-06

**Authors:** Saeid Najafi-Fard, Elisa Petruccioli, Chiara Farroni, Linda Petrone, Valentina Vanini, Gilda Cuzzi, Andrea Salmi, Anna Maria Gerarda Altera, Assunta Navarra, Tonino Alonzi, Emanuele Nicastri, Fabrizio Palmieri, Gina Gualano, Valentina Carlini, Douglas McClain Noonan, Adriana Albini, Delia Goletti

**Affiliations:** ^1^ Translational Research Unit, National Institute for Infectious Diseases Lazzaro Spallanzani- Istituto di Ricovero e Cura a Carattere Scientifico (IRCCS), Rome, Italy; ^2^ Department of Epidemiology and Preclinical Research, UOS Professioni Sanitarie Tecniche National Institute for Infectious Diseases Lazzaro Spallanzani-Istituto di Ricovero e Cura a Carattere Scientifico (IRCCS), Rome, Italy; ^3^ Clinical Epidemiology Unit, National Institute for Infectious Disease Lazzaro Spallanzani-Istituto di Ricovero e Cura a Carattere Scientifico (IRCCS), Rome, Italy; ^4^ Clinical Division of Infectious Diseases, National Institute for Infectious Diseases Lazzaro Spallanzani-Istituto di Ricovero e Cura a Carattere Scientifico (IRCCS), Rome, Italy; ^5^ Respiratory Infectious Diseases Unit, National Institute for Infectious Diseases Lazzaro Spallanzani-Istituto di Ricovero e Cura a Carattere Scientifico (IRCCS), Rome, Italy; ^6^ Unit of Molecular Pathology, Biochemistry and Immunology, Istituto di Ricovero e Cura a Carattere Scientifico (IRCCS) MultiMedica, Milan, Italy; ^7^ Immunology and General Pathology Laboratory, Department of Biotechnology and Life Sciences, University of Insubria, Varese, Italy; ^8^ European Institute of Oncology IEO-Istituto di Ricovero e Cura a Carattere Scientifico (IRCCS), Milan, Italy

**Keywords:** COVID-19, SARS-CoV-2, spike, IL-10, whole-blood, Natutal Killer Cells, T cell, cytokine

## Abstract

**Objective:**

Several therapies with immune-modulatory functions have been proposed to reduce the overwhelmed inflammation associated with COVID-19. Here we investigated the impact of IL-10 in COVID-19, through the *ex-vivo* assessment of the effects of exogenous IL-10 on SARS-CoV-2-specific-response using a whole-blood platform.

**Methods:**

Two cohorts were evaluated: in “study population A”, plasma levels of 27 immune factors were measured by a multiplex (Luminex) assay in 39 hospitalized “COVID-19 patients” and 29 “NO COVID-19 controls” all unvaccinated. In “study population B”, 29 COVID-19 patients and 30 NO COVID-19-Vaccinated Controls (NO COVID-19-VCs) were prospectively enrolled for the IL-10 study. Whole-blood was stimulated overnight with SARS-COV-2 antigens and then treated with IL-10. Plasma was collected and used for ELISA and multiplex assay. In parallel, whole-blood was stimulated and used for flow cytometry analysis.

**Results:**

Baseline levels of several immune factors, including IL-10, were significantly elevated in COVID-19 patients compared with NO COVID-19 subjects in “study population A”. Among them, IL-2, FGF, IFN-γ, and MCP-1 reached their highest levels within the second week of infection and then decreased. To note that, MCP-1 levels remained significantly elevated compared with controls. IL-10, GM-CSF, and IL-6 increased later and showed an increasing trend over time. Moreover, exogenous addition of IL-10 significantly downregulated IFN-γ response and several other immune factors in both COVID-19 patients and NO COVID-19-VCs evaluated by ELISA and a multiplex analysis (Luminex) in “study population B”. Importantly, IL-10 did not affect cell survival, but decreased the frequencies of T-cells producing IFN-γ, TNF-α, and IL-2 (p<0.05) and down-modulated HLA-DR expression on CD8^+^ and NK cells.

**Conclusion:**

This study provides important insights into immune modulating effects of IL-10 in COVID-19 and may provide valuable information regarding the further *in vivo* investigations.

## Introduction

The Coronavirus Disease 2019 (COVID-19) caused by severe acute respiratory syndrome coronavirus 2 (SARS-CoV-2) is characterized by excessive production of pro-inflammatory cytokines and acute lung damage associated with patient mortality (1, 2). Hyperactivation of the immune system results in an acute increase in circulating levels of pro-inflammatory cytokines, bringing to a cytokine storm that can lead to acute respiratory distress syndrome (ARDS), multiorgan failure, and death (3). Profound alterations in the innate and adaptive immune compartments such as neutrophilia, lymphopenia, and altered lymphocyte function have been reported in SARS-CoV-2 infection (1). Activated antigen-specific T cells produce a variety of effector molecules for clearing infection, but also significantly contribute to inflammation and tissue injury (4). Levels of circulating factors including interleukin (IL)-6, IL-18, Interferon (IFN)-γ, IL-15, Tumor Necrosis Factor (TNF)-α, IL-1α, IL-1β, IFN-γ-inducible protein (IP-10), and IL-2 are significantly elevated in patients with moderate or severe COVID-19 and particularly in the fatal course of disease (3, 5).

Although large-scale vaccine administration is available in the majority of countries, the identification of new effective therapies is still crucial for unvaccinated or vaccinated vulnerable subjects at higher risk to develop severe disease (6). Moreover, due to the emergence of new variants of concern (VOC) evading the immune protection mediated by the vaccine, there is an urgent need to develop new therapeutic strategies (7) especially involving T cells being more stable over time (8) and recognizing the VOC in both immune competent (9) and immune deficient individuals (10, 11).

Patients with severe disease show lower levels of CD4^+^ and CD8^+^ T cells and higher levels of pro-inflammatory plasma IL-6 compared with patients with mild illness, associated with reduced patient survival, highlighting the important role of this immune mediator in the pathogenesis and COVID-19 severity (12). Several trials with IL6 inhibitors drugs, such as tocilizumab, have been performed in the early times of the epidemic. However, large evidence shows also an increase of interleukin IL-10 in COVID-19 patients, which can be considered a crucial feature of COVID-19 (12).

Few investigations are available on the action of IL-10 in COVID-19. IL-10 plays a critical role in the resolution of peripheral inflammation. It is produced by a variety of immune cells including activated macrophages, Th1, Th2, Th17, and T-reg cells (13) and it is a key anti-inflammatory cytokine reducing the expression of inflammatory cytokines such as IL-1β, IL-6, and TNF-α. After interaction with its receptor, IL-10 activates the JAK1-TYK2-STAT3 pathway leading to STAT3-mediated transcription of genes that limit the inflammatory response (14). In addition, IL-10 can suppress the activity of the fibroblasts acting as a potent anti-fibrotic agent reducing the pulmonary fibrosis (15, 16). Moreover, we have reported that IL-10 may increase ACE2 expression in the lung-derived Calu-3 cell line and endothelial cells in a dose-dependent manner, suggesting a potential role of IL-10 in SARS-CoV-2-associated clinical outcome (17).

Several studies have shown that ACE2/Angiotensin- (1–7)/Mas axis reduces cytokine release and inhibits signaling pathways of inflammation (17, 18). Rapid accumulation of proinflammatory cytokines in patients with severe disease can strongly stimulate IL-10 production as a negative feedback loop (1) to suppress hyperinflammation and prevent tissue damage (14). This may be the clinical significance of excessive production of IL-10 in the serum of COVID-19 patients. However, the late onset of IL-10 production may be inadequate to limit the elevated secretion of inflammatory immune factors and activation of proinflammatory cells in COVID-19 (1).

Currently, several therapies are used for the COVID-19 treatment to reduce the overwhelmed inflammation and the SARS-CoV-2-mediated activation, such as IL-1β inhibitors, IL-6 inhibitors, JAK-inhibitors, and corticosteroids (19–21). Interestingly, based on its immunoregulatory function, administration of IL-10 has been proposed to treat ARDS in COVID-19 (1). IL-10 agonists have also been developed for other inflammatory diseases (22). However, a more comprehensive study is required to define both the potential protective and pathological roles of IL-10 in COVID-19 immune-pathogenesis (12).

Here, we investigated *ex-vivo* the effect of IL-10 on the SARS-CoV-2-specific and -unspecific response of COVID-19 patients (with or without vaccination) and in vaccinated controls, using a whole-blood platform (23–25). We also characterized its effect at a cellular level by flow cytometry.

## Materials and methods

### Study population

The present study was approved by the Ethical Committee of Lazzaro Spallanzani National Institute of Infectious Diseases (59/2020, 72/2015, 247/2021, 297/2021) and was conducted between April 15, 2020 and March 22, 2022.

To perform the multiplex analysis for evaluation of immune-factors at baseline, we prospectively enrolled (April 15, 2020- September 2, 2021) 68 individuals all unvaccinated against COVID-19 (study population “A”) including 29 “NO COVID-19” and 39 acute hospitalized COVID-19 patients (26) (**Table 1**). Inclusion criteria for COVID-19 patients were a diagnosis based on a positive nasopharyngeal swab for SARS-CoV-2 and a disease with specific clinical characteristics (21, 25) evaluated at the highest disease. Within COVID-19 patients 9 were individuals with tuberculosis infection (TBI). “NO-COVID-19”-controls were healthy donors (n=14) and individuals with tuberculosis infection (27) (n=15), with no symptoms of COVID-19 and a negative SARS-CoV-2 serology and/or a negative swab for SARS-CoV-2.

**Table 1:** Demographical and clinical characteristics of study population “A” for baseline evaluation of cytokines, chemokines, and growth factors in plasma.

		COVID-19	NO COVID-19	Total	*p*
N (%)		39 (57.3)	29 (42.7)	68	–
Age median (IQR)		54 (44-64)	56 (38.5-61.5)	55 (41.75-63.75)	0.9282*
Male N (%)		26 (66.7)	14 (48.3)	40 (58.8)	0.1436^§^
Origin N (%)	West Europe	28 (71.8)	24 (82.8)	52 (76.5)	0.0840^§^
	East Europe	0 (0)	2 (6.8)	2 (2.9)	
	Asia	7 (18)	0 (0)	7 (10.3)	
	Africa	1 (2.5)	1 (3.6)	2 (2.9)	
	South America	3 (7.7)	2 (6.8)	5 (7.4)	
COVID-19 vaccination status N (%)	Unvaccinated Vaccinated	39 (100) 0	29 (100) 0	68 (100) 0	–
Swab positive results N (%)		39 (100)	0 (0)	–	na
Severity N (%) ^#^	Mild	7 (18)	–	–	
	Moderate	19 (48.7)	–	–	na
	Severe	8 (20.5)	–	–	
	Critical	5 (12.8)	–	–	
Days post symptoms onset N (%)	0-7	16 (41)	–	–	
	8-14	13 (33.4)	–	–	na
	15-21	8 (20.5)			
	22-31	2 (5.1)	–	–	

COVID-19, COronaVIrus Disease 19; N, Number; *Mann Whitney test; ^§^ Chi-square test; ^#^ WHO criteria, ref (26); na, not applicable.

To evaluate the effect of IL-10 on the SARS-CoV-2 specific immune response, we prospectively enrolled (November 8, 2021-March 30, 2022) a second study population (study population “B”) including 30 “NO COVID-19-vaccinated controls” (NO COVID-19-VC) and 29 hospitalized COVID- 19-patients (**Table 2** and **Figure S1**). Within COVID-19 patients, 2 concomitantly had active tuberculosis (TB), one had TBI, and one had lymphoma. Vaccinated controls were healthy donors (n=11), subjects with: TBI (n=10), TBI and rheumatological disease (n=1), TBI and multiple sclerosis (n=2), rheumatoid arthritis (n=1), TB (n=4), TB and rheumatoid arthritis (n=1). The study complied with the principles of the Declaration of Helsinki.

**Table 2:** Demographical and clinical characteristics of the study population “B” for evaluating the effect of IL-10 on the SARS-CoV-2 specific immune response.

		COVID-19	NO-COVID-19-VC	Total	*p*
N (%)		29 (49.2)	30 (50.8)	59	–
Age median (IQR)		67 (50.5-76.5)	46.5 (34.25-55.25)	53 (43-72)	0<0.0001*
Male N (%)		18 (62.1)	12 (40)	30 (50.8)	0.120^§^
COVID-19 Vaccination status N (%)	Unvaccinated Vaccinated	15 (51.7) 14 (48.3)	0 (0) 30 (100)	15 (25.4) 44 (74.6)	
Origin N (%)	West Europe	24 (82.8)	19 (63.3)	43 (73)	0.171^§^
	East Europe	3 (10.3)	5 (16.7)	8 (13)	
	Asia	2 (6.9)	1 (3.3)	3 (5)	
	Africa	0 (0)	4 (13.4)	4 (7)	
	South America	0 (0)	1 (3.3)	1 (2)	
Swab positive results N (%)		29 (100)	0 (0)	–	na
Spike Responders N (%)	Total	14 (48.3)	25 (83.3)	39 (66)	0.0061^§^
	COVID-19 vaccinated	8 (57.1)^a^	25 (100)^a^	33 (84)^a^	0.0009^§^
Severity N (%) ^#^	Moderate	14 (48.3)	–	–	na
	Severe	12 (41.4)	–	–	
	Critical	3 (10.3)	–	–	

COVID-19, COronaVIrus Disease 19; N, Number; NO-COVID-19 VC, NO-COVID-19 Vaccinated Control; *Mann Whitney test; ^§^ Chi-square test; ^#^ WHO criteria, ref (26); ^a^ percentage calculated on the number of Spike responders; na, not applicable.

### Peptide pools and stimuli

SARS−CoV-2 PepTivator ^®^ Peptide Pool (PepTivator^®^ SARS-CoV-2 Prot_S1, Prot_S, and Prot_S+) of the spike protein (Pool S) (Miltenyi, Biotec, Germany) was used for whole-blood stimulation. The PepTivator^®^ Peptide Pools are constituted by peptides of 15 amino acid length with 11 amino acid overlap. The PepTivator^®^ SARS-CoV-2 Prot_S covers selected immunodominant sequence domains of the spike protein (aa 304–338, 421–475, 492–519, 683–707, 741–770, 785–802, and 885–1273). The PepTivator^®^ SARS-CoV-2 Prot_S1 covers the N-terminal S1 domain of the spike protein (aa 1–692). The PepTivator^®^ SARS-CoV-2 Prot_S+ covers the gaps in the sequence mapping between aa 689 and 895 of the PepTivator^®^ SARS-CoV-2 Prot_S (24).

### IL-10 cytokine

IL-10 cytokine was purchased from Miltenyi Biotec (Bergisch Gladbach, Germany). To assess the role of IL-10 on the modulation of immune responses, we evaluated the effect of IL-10 at different concentrations (1, 5, and 10 ng/ml) on the modulation of spike-specific and Staphylococcal Enterotoxin B (SEB) induced cell response.

### SARS−CoV−2 serology

SARS-CoV-2 specific IgM and IgG levels were measured by enzyme-linked immunosorbent assay (ELISA) according to manufacturer’s instructions (DIESSE Diagnostica Senese S.p.a., Monteriggioni, Italy). The ratio between the optical density (OD) of the sample and that one of the cut-off reagent (index) was calculated. The samples were scored positive (index > 1.1), doubtful (index between 1.1 and 0.9) and negative (index < 0.9).

### Whole-blood assay

In a 48-well flat-bottom plate whole-blood was stimulated or not with the described Pool S (spike) at 0.1 μg/mL and SEB (Sigma- Aldrich, St. Loius, MO, USA) at 200 ng/mL (positive control) and then treated or not with recombinant human IL-10. The plate was incubated overnight (20–24 h) at 37°C, 5% CO_2_, and then plasma was collected and stored at − 80°C until use (24, 28).

### IFN-γ detection

IFN-γ levels were evaluated by ELISA, according to manufacturer’s instructions (www.quantiFERON.com). IFN-γ value was subtracted from the unstimulated control. The lower detection limit of the kit was 0.065 IU/ml.

### Multiplex analysis

Cytokines, chemokines and growth factors including IL-1β, interleukin-1 receptor antagonist (IL-1RA), IL-2, IL-4, IL-5, IL-6, IL-7, IL-8, IL-9, IL-10, IL-12p70, IL-13, IL-15, IL-17A, Eotaxin, fibroblast growth factor (FGF)-basic, granulocyte colony stimulating factor (G-CSF), granulocyte macrophage colony-stimulating factor (GM-CSF), IFN-γ, IP-10, monocyte chemoattractant protein-1 (MCP-1), macrophage inflammatory protein (MIP)-1α, MIP-1β, Platelet-derived growth factor (PDGF), regulated on activation, normal T cell expressed and secreted (RANTES), TNF-α, and vascular endothelial growth factor (VEGF) were evaluated using a Luminex Bio-Plex Pro Human Cytokine 27-plex assay panel and the MagPix system (Bio-Rad, Hercules, CA, USA). Raw data were generated using the Bio-Plex manager software. Concentrations below the detection range were considered as zero. Concentrations above the detection range were converted to the highest value of the standard curve. Analyte levels were subtracted from the unstimulated control. Samples with acquired beads count <50 were excluded from the final analysis (25).

### Functional analysis by intracellular staining and flow cytometry

Briefly, whole-blood was stimulated or not with the described Pool S (spike) at 0.1 μg/mL and SEB (Sigma- Aldrich, St. Loius, MO, USA) at 200 ng/mL (positive control) and then treated or not with recombinant human IL-10. After 1 hour, brefeldin A (10 µg/ml) (Life Technologies, Monza, Italy) was added to inhibit cytokine secretion (23) and the plate was incubated overnight (20–24 h) at 37°C, 5% CO2. Next, blood was harvested and stained with Fixable Viability stain 700 (BD Biosciences, San Jose, USA) for 10 min at room temperature (RT) protected from light. Red blood cells were then lysed with BD Lysing Solution (BD Biosciences, San Jose, USA) + 4% of formaldehyde for 10 min at RT, and then cells were washed with 1 ml of phosphate-buffered saline (PBS) and centrifuged at 600 × g for 5 min. After that, cells were fixed with 4% formaldehyde for 5 min, washed again with 1 ml of PBS, centrifuged at 600 × g for 5 min, and frozen in fetal bovine serum (FBS) + 10% dimethyl sulfoxide (DMSO) until further use. Stimulated and fixed cells were thawed at 37°C, washed twice with PBS at 600 × g for 5 min and transferred to a 96-well round plate (COSTAR, Sigma Aldrich), and stained for the surface and intracellular markers with the following antibodies: CD4-ECD (Beckman Coulter), CD3-V450, CD8-APC-H7, CD16-PerCP-Cyanine5.5, CD56-APC, HLA-DR-BV786, IFN-γ-BV510, TNF-α-FITC (all from BD Biosciences), and IL-2-PE (Miltenyi). At the end of the procedure, samples were washed twice in “Perm/Wash” buffer (BD Biosciences), acquired using a DxFLEX cytometer and analyzed with FlowJo software (version 10.8.1, Tree Star). A specific response was considered as positive when the percentage of the stimulated population was at least 2-fold higher compared to the unstimulated control with a minimum of 10 events presented in the cytokine gate (24). Gating strategy is shown in **Figure S2**.

### Statistical analysis

Data were analyzed using Graph Pad (GraphPad Prism 8 XML ProjecT) and Stata (StataCorp. 2021. Stata Statistical Software: Release 17. College Station, TX: StataCorp LLC). Medians, interquartile ranges (IQRs) were used to summarize all the analysis with the exception of the results shown as heat map where each immune factor value was normalized by subtracting the mean cytokine value calculated for each specific cytokine within each group. Subsequently, this value was divided by the standard deviation calculated for the specific cytokine within each group. Mann Whitney U test for comparisons among groups; Chi-squared test for categorical variables and Wilcoxon matched-pairs signed rank test to assess differences between the paired samples were used. We also evaluated the relative variation after IL-10 treatment expressed as percentage and to overcome problems with baseline zeros, all zeroes were replaced with a small value (i.e. 0.00001). One-sample Wilcoxon signed-rank tests were used to evaluate which analyte varied from the overall median change within each group. P-values <0.05 were considered statistically significant.

## Results

### Demographic and clinical characteristics of the studied populations

Demographical and clinical information of the enrolled subjects are shown in **Table 1** and **Table 2**. Subjects of study population “A” were not vaccinated against SARS-CoV2. They were mostly male and from Western Europe. Most of COVID-19 patients had moderate and mild disease (**Table 1**).

Subjects of study population “B” included vaccinated individuals were equally distributed as male and female and mostly from Western Europe. The 48.3% of COVID-19 was vaccinated against SARS-CoV-2 and had moderate disease. About half of COVID-19 patients and 83% of NO COVID-19-VC subjects responded to the *in vitro* spike stimulation, based on an already defined cut-off (0.13 IU/mL) (24). To note, among the COVID-19 patients responding to spike stimulation, the 57% were vaccinated against SARS-CoV-2 (**Table 2** and **Figure S1**). The days post-vaccination was available for 8/14 (57.1%) COVID-19 patients (71 days after Johnson & Johnson for one patient, 119-257 days post the second dose of BNT162b2 for 6 patients, 34 days post booster dose of BNT162b2 for one patient, and 10 days post mRNA-1273 booster dose for one patient). Most of spike responders (6/8, 75%) had received at least 2 doses of a vaccine against SARS-CoV-2. Half of NO COVID-19-Vaccinated subjects had received at least 2 doses of either a viral vector-based or an mRNA vaccine (21-272 days post vaccination) and the rest had received the booster (23-77 days post booster).

### Plasma levels of several immune factors are significantly increased in COVID-19 patients

We evaluated the levels [picograms per milliliter (pg/mL)] of several cytokines and chemokines and growth factors in plasma of subjects from study population “A”. Several pro-inflammatory cytokines and chemokines including IL-1β, IL-6, IL-17A, IFN-γ, IP-10, MCP-1, RANTES, TNF-α, anti-inflammatory cytokines including IL-4 and IL-10, and growth factors including IL-2, IL-5, FGF-basic, G-CSF, and GM-CSF were significantly elevated in COVID-19 patients compared to “NO-COVID-19” subjects. Interestingly, beside the pro-inflammatory cytokines and chemokines, IL-10 was significantly associated with COVID-19 status (**Table 3**). In addition, within “NO-COVID-19” group, no significant difference was found in cytokines’ levels between healthy donors (n=14) and TBI (n=15) subjects (data not shown).

**Table 3:** The baseline plasma level of cytokines, chemokines, and growth factors found significantly elevated in COVID-19 patients compared to NO COVID-19 subjects of study population “A”.

Function	Main Source	Analyte	COVID-19 Median (IQR)	NO COVID-19Median (IQR)	P*
Pro-Inflammatory cytokine/chemokine	activated macrophages	IL-1β	2.44 (1.40-4.64)	1.16 (0.62-2.96)	0.0076
	Macrophages	IL-6	12.88 (6.64-33.84)	6.48 (3.68-16.98)	0.0039
	Th17	IL-17A	12.60 (7.12-19.32)	7.88 (5.02-10.56	0.0038
	Th1	IFN-γ	123.5 (63.92-277)	34.44 (17.46-87.22)	<0.0001
	Monocytes, Fibroblasts, Endothelial Cells	IP-10	1406 (590.4-2611)	444 (313.8-942.4)	0.0042
	Monocytes, Macrophages	MCP-1	1967 (979.1-4928)	313.7 (199-768.6)	<0.0001
	Platelets, Macrophages	RANTES	4153 (3079-8045)	2711 (1911-5489)	0.0241
	Macrophages	TNF-α	63.52 (49.24-93.04)	48.64 (31.66-63.94)	0.010
Anti-inflammatory cytokine	Th2	IL-4	2.08 (1.28-3.64)	1.56 (0.58-2.80)	0.043
	Th2, Treg	IL-10	6.68 (4.52-11.60)	3.56 (2.06-6.98)	0.0014
Growth factor	Th1	IL-2	5.72 (2.72-9.68)	2.52 (0.48-5.04)	0.0012
	Th2, Mast cells	IL-5	37.96 (4.00-61.28)	5.8 (0.0-35.4)	0.0066
	Stromal cells, Macrophages	FGF-basic	49.04 (32.88-68.80)	28.68 (14.36-38.58)	0.0004
	endothelium, macrophages,	G-CSF	429.3 (164.9-650.3)	197.8 (134.9-357.2)	0.0411
	macrophages, T cells, mast cells, NK cells	GM-CSF	2.52 (0.28-4.76)	0.32 (0.0-2.30)	0.0015

COVID-19, CoronaVIrus Disease 19; * Mann Whitney test; IQR, Interquartile Range; IL, interleukin; IFN, interferon; IP, IFN-γ inducible Protein; MCP, Monocyte Chemoattractant Protein; RANTES, Regulated upon Activation, Normal T Cell Expressed and Presumably Secreted; TNF, Tumor Necrosis Factor; FGF, Fibroblast Growth Factor; G-CSF, Granulocyte-Colony Stimulating Factor; GM-CSF, Granulocyte-Macrophage-Colony-Stimulating Factor; VEGF, Vascular-Endothelial Growth Factor.

**Figure 1:**
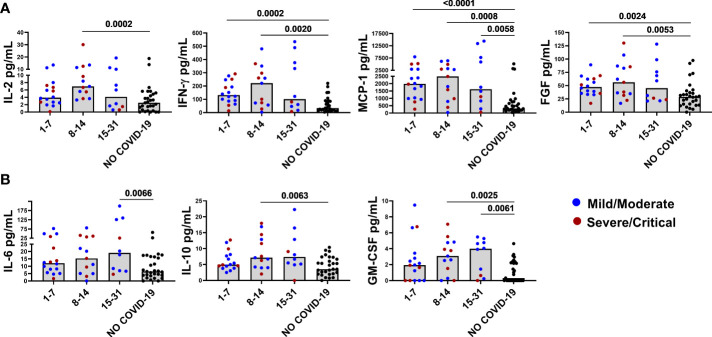
Baseline levels of elevated immune markers based on the days post symptoms onset. Panel **(A)** IL-2, IFN-γ, FGF, and MCP-1 reached their highest levels within the second week of infection and then decreased by week 3. Panel **(B)** IL-6, IL-10, and GM-CSF increased later and showed an increasing trend over time. After correction for multiple comparisons, p≤ 0.008 was considered as significant.

We then analyzed the levels of elevated immune factors according to the days post symptoms onset and the disease severity. IL-2, FGF, IFN-γ, and MCP-1 reached their highest levels within the second week of infection and then decreased. To note that, the MCP-1 levels remained significantly elevated compared with controls (**Figure 1A**). Compared with controls, the FGF, IFN-γ, and MCP-1 levels were already significantly higher at week 1. On the other hand, IL-10, GM-CSF, and IL-6 increased later and showed an increasing trend over time. Within the second week, levels of IL-10 and GM-CSF were significantly higher compared with NO COVID-19 controls and remained elevated. IL-6 reached significance within 15-31 days post symptoms onset (**Figure 1B**).

**Figure 2:**
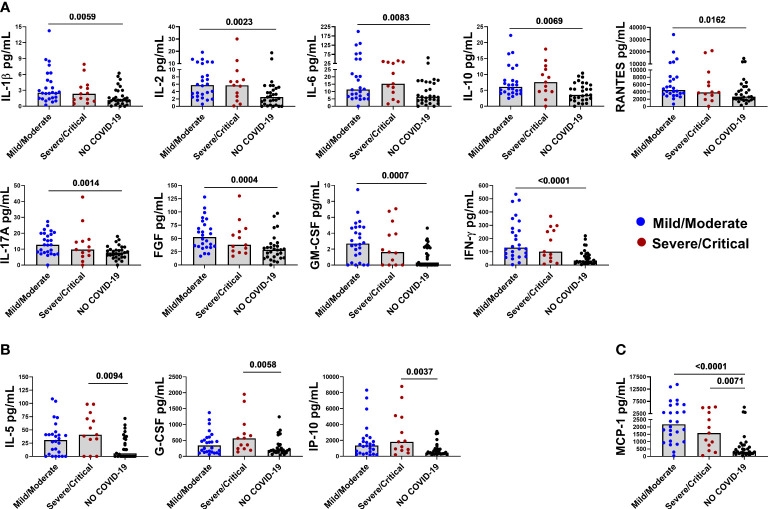
Baseline levels of elevated immune markers based on the disease severity. Panel **(A)** IL-1β, IL-2, IL-6, IL-10, RANTES, IL-17A, FGF-basic, GM-CSF, and IFN-γ are associated with mild/moderate disease. Panel **(B)** IL-5, G-CSF, and IP-10 are associated with severe/critical disease. Panel **(C)** MCP-1 is associated with both mild/moderate and severe/critical disease. After correction for multiple comparisons, p≤ 0.016 was considered as significant.

Furthermore, IL-1β, IL-2, IL-6, IL-10, IL-17A, RANTES, FGF-basic, GM-CSF, and IFN-γ were found to be associated with mild/moderate disease (**Figure 2A**), whereas IL-5, G-CSF, and IP-10 were associated with severe/critical disease (**Figure 2B**). Interestingly, MCP-1 was associated with COVID-19 disease per se because it was increased in both mild/moderate and severe/critical disease (**Figure 2C**) compared with controls.

### IL-10 significantly downregulates spike-induced IFN-γ response

**Figure 3:**
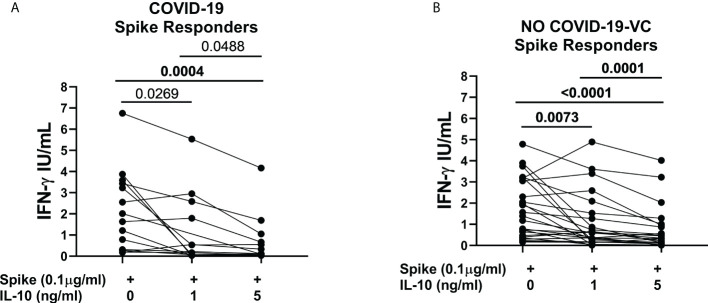
IL-10 significantly downregulates Spike-induced IFN-γ response in COVI-19 patients and NO COVID-19-Vaccinated Controls (NO COVID-19-VC) of study population “B”. **(A)** COVID-19 patients, responders to Spike stimulation (n=14), **(B)** NO COVID-19-VCs, responders to Spike stimulation (n=25). IFN-γ production in response to Spike (Pool S) was considered positive based on a cut-off (0.13 IU/mL) defined in our previous study. After correction for multiple comparisons, p≤ 0.016 was considered as significant.

In the study population “B”, we *ex-vivo* evaluated the immunomodulatory effect of IL-10 at different concentrations (1, 5, and 10 ng/ml) on the SARS-CoV-2-specific peripheral blood cells responses in COVID-19 patients and NO COVID-19-VC subjects. Importantly, no significant difference was detected between the results obtained using IL-10 at 5 ng/ml and at 10 ng/ml neither in response to spike nor to SEB in a cohort of 9 controls (**Figure S3**). Therefore, hereafter we used IL-10 at a concentration of 5 ng/ml. We then found that IL-10 at 5 ng/ml significantly downregulates spike-induced IFN-γ response in spike-responders of COVID-19 patients (14/29) [1.82 (0.30-3.468) vs 0.11 (0.37-0.75), p=0.0004] identified based on a cut-off previously defined (24) (**Figure 3A**).

Moreover, IL-10 significantly downregulated the spike-induced IFN-γ response in spike-responders of NO-COVID-19-VCs (25/30) [1.39 (0.42-3.11) vs 0.33 (0.12-0.91), p<0.0001] (**Figure 3B**). IL-10 also reduced SEB-induced IFN-γ response in the spike-responders of COVID-19 patients [15.21 (12.12-17.35) vs 7.52 (1.94-16.22), p=0.0067] (**Figure S4A**); as well as in NO COVID-19-VCs, although the difference of SEB-induced IFN-γ response did not reach significance (p>0.016) (**Figure S4B**).

### IL-10 significantly downregulates several spike-induced immune factors

**Figure 4:**
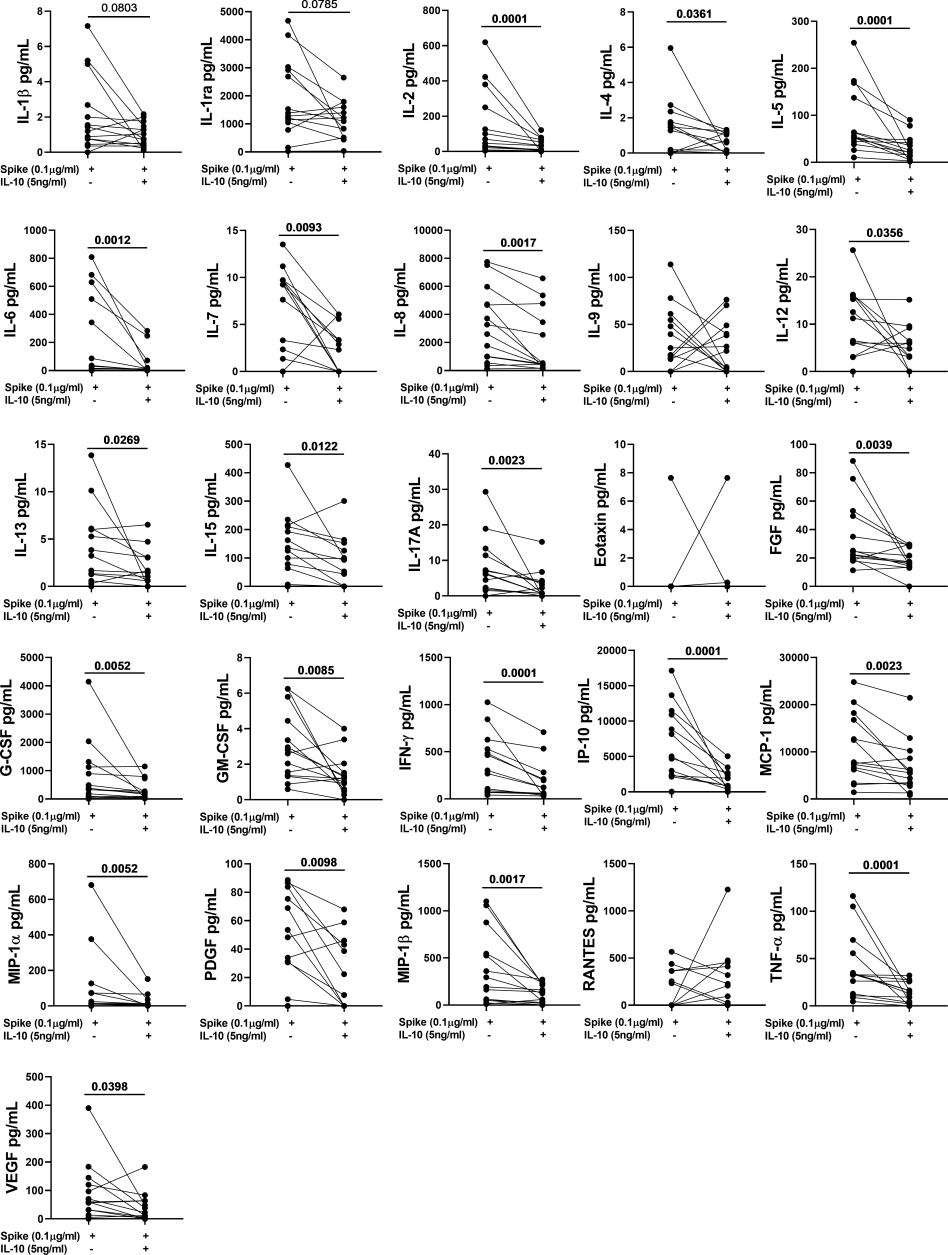
IL-10 significantly downregulates several Spike-induced cytokines, chemokines, and growth factors in COVID-19 Patients of study population “B”. Whole-blood was stimulated or not with a pool of Spike peptides (Pool S) of SARS-COV-2 and then treated or not with IL-10. After overnight stimulation, plasma was collected and used to detect immune factors by a 27-plex multiplex assay (n=14).

**Figure 5:**
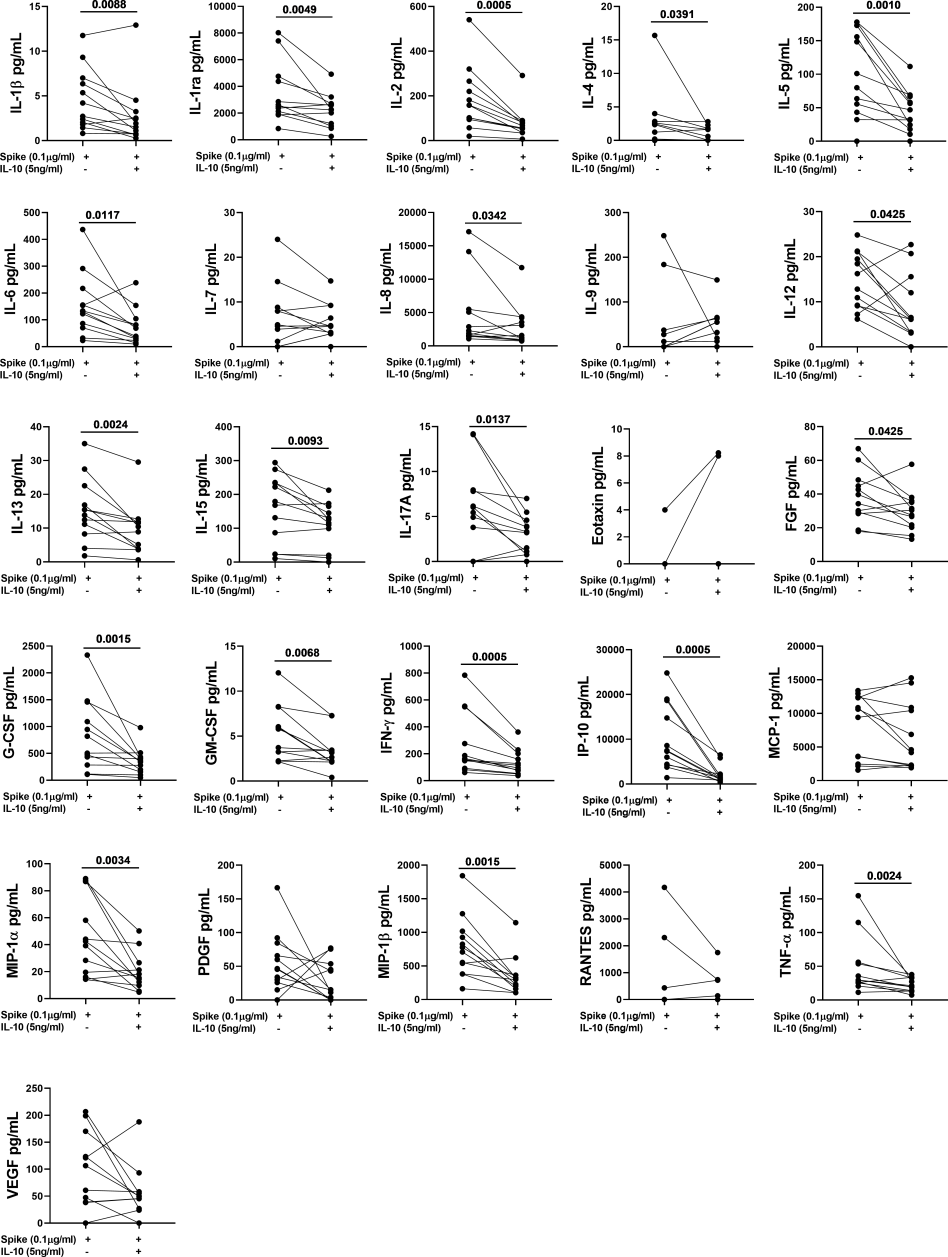
IL-10 significantly downregulates several Spike-induced cytokines, chemokines, and growth factors in NO COVID-19-Vaccinated Controls (NO COVID-19-VC) of study population “B”. Whole-blood was stimulated or not with a pool of Spike peptides (Pool S) of SARS-COV-2 and then treated or not with IL-10. After overnight stimulation, plasma was collected and used to detect immune factors by a 27-plex multiplex assay (n=12).

To better characterize the effect of exogenous IL-10 on the whole-blood cells response after stimulation, we measured 27 different immune factors by Luminex multiplex technology in 26 subjects including 14 COVID-19 patients and 12 NO COVID-19-VCs of study population “B”. IL-10 significantly downregulated the SARS-CoV-2 specific response of several cytokines, chemokines, and growth factors within both COVID-19 patients (**Figure 4**, **Table 4**) and NO-COVID-19-VCs (**Figure 5**, **Table 4**) . A heatmap summarizing the results is shown in the **Figure S5**. Moreover, a similar trend was observed in SEB-induced immune factors production in COVID-19 Patients (**Figure S6**, **Table S1**) and in NO-COVID-19-VCs (**Figure S7**, **Table S1**). A heatmap of the evaluated factors for the effect of IL-10 on SEB-induced response in COVID-19 patients and NO-COVID-19-VC is shown in the **Figure S8**.

**Table 4:** IL-10 significantly downregulates several Spike-induced cytokines, chemokines, and growth factors in COVID-19 patients and NO COVID-19-VCs of study population “B”, using a 27-plex multiplex assay.

		COVID-19 Patients (n=14)			NO-COVID-19-VC (n=12)		
Function	Analyte	Spike Median (IQR)	Spike + IL-10 Median (IQR)	P*	Spike Median (IQR)	Spike + IL-10 Median (IQR)	P*
Pro-Inflammatory cytokine/chemokine	**IL-1β**	1.32 (0.68-3.26)	0.9 (0.42-1.55)	0.0803	3.46 (1.86-6.84)	1.72 (0.76-3.06)	0.0088
	**IL-6**	33.6 (8.77-540.6)	9.72 (2.49-33.03)	0.0012	132.1 (74.42-201.8)	53.94 (25.03-98.74)	0.0117
	**IL-8**	2945 (877.5-5040)	489.3 (357.4-3784)	0.0017	2084 (1492-5383)	1417 (879.9-4049)	0.0342
	**IL-12**	8.88 (3.10-15.96)	4.04 (0-7.14)	0.0356	14.52 (9.15-20.71)	6.12 (3.13-14.67)	0.0425
	**IL-17A**	6.68 (2.24-11.89)	1.52 (0.76-3.98)	0.0023	5.74 (0.95-7.92)	3.26 (0.84-4.43)	0.0137
	**IFN-γ**	285.1 (71.1-553.1)	90.74 (47.96-227.9)	0.0001	159.9 (106.5-481.3)	105.4 (59.7-191.7)	0.0005
	**IP-10**	4860 (2229-11044)	862.4 (277.8-2422)	0.0001	7395 (4310-17682)	1308 (908.7-2109)	0.0005
	**MCP-1**	7664 (5507-17151)	5525 (3087-9017)	0.0023	10024 (2741-12388)	4382 (2246-10765)	0.176
	**MIP-1α**	14.4 (8.11-86.84)	7.62 (2.28-19.51)	0.0052	40.92 (16.95-79.66)	16.68 (10.42-25.27)	0.0034
	**MIP-1β**	243.8 (50.65-628.4)	132.3 (26.62-226.5)	0.0017	741.2 (421.7-995)	297.8 (170.3-354.1)	0.0015
	**TNF-α**	33.18 (12.34-59.19)	14.66 (3.39-25.88)	0.0001	29.48 (25.58-55.53)	20.44 (13.55-33.35)	0.0024
Anti-inflammatory cytokine	**IL-1rα**	1335 (984.4-2950)	1154 (512.1-1644)	0.0785	2557 (1970-4666)	2259 (1078-2700)	0.0049
	**IL-4**	1.38 (0-1.91)	0.38 (0-1.10)	0.0361	1.8 (0.0-2.78)	0.32 (0.0-1.76)	0.0391
	**IL-13**	2.46 (0.53-6.03)	1.24 (0-3.05)	0.0269	14.64 (8.998-21.13)	9.68 (3.86-11.8)	0.0024
Growth factor	**IL-2**	57.98 (2.38-282.9)	22.73 (5.19-70.53)	0.0001	158.6 (96.49-254.4)	61.62 (40.03-85.68)	0.0005
	**IL-5**	54.5 (42.82-145.3)	23.94 (11.21-43.36)	0.0001	90.5 (46.33-168.7)	39.68 (19.5-64.54)	0.001
	**IL-7**	8.44 (1.02-9.72)	1.16 (0-3.92)	0.0093	4.76 (0.3-8.59)	4.58 (2.88-8,53)	0.505
	**IL-15**	131 (49.98-212.1)	73.02 (0-132.9)	0.0122	173.6 (39.32-234.8)	120.8 (39.79-160)	0.0093
	**FGF-basic**	23.5 (19.15-50.44)	16.9 (14.07-28.42)	0.0039	37.02 (28.64-47.54)	28.96 (20.67-35.74)	0.0425
	**G-CSF**	359.3 (94.81-1178)	192.6 (55.72-384.9)	0.0052	662.4 (322.1-1366)	348.6 (164.9-425.7)	0.0015
	**GM-CSF**	2.7 (1.36-4.78)	1.21 (0.57-1.65)	0.0085	4.76 (2.49-7.69)	2.90 (2.13-3.43)	0.0068
	**PDGF**	41.14 (3.54-77.49)	3.84 (0-43.88)	0.0098	46.06 (26.97-79.84)	15.16 (0.98-51.68)	0.176
	**VEGF**	59.38 (27.18-126.3)	16.96 (3.75-63.61)	0.0398	83.82 (38.5-158.6)	47.28 (24.85-57.58)	0.147

COVID-19, CoronaVIrus Disease 19; * Wilcoxon matched-pairs signed rank test; NO COVID-19-VC, NO-COVID-19-Vaccinated Control; IQR, Interquartile Range; IL, interleukin; IFN, interferon; IP, IFN-γ inducible Protein; MCP, Monocyte Chemoattractant Protein; MIP, Macrophage Inflammatory Protein; TNF, Tumor Necrosis Factor; FGF, Fibroblast Growth Factor; G-CSF, Granulocyte-Colony Stimulating Factor; GM-CSF, Granulocyte-Macrophage-Colony Stimulating Factor; PDGF, Platelet-Derived Growth Factor; VEGF, Vascular-Endothelial Growth Factor.

**Figure 6:**
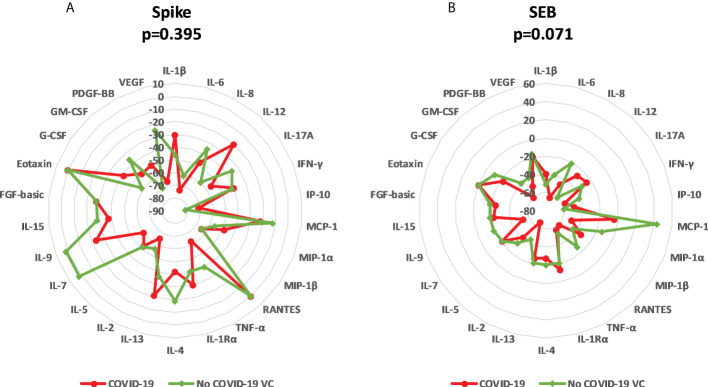
Modulation of immune factors after IL-10 treatment for each analyte in COVID-19 patients and NO COVID-19-Vaccinated Controls (NO COVID-19-VC) of study population “B”. **(A)** Spike stimulation; **(B)** SEB stimulation. Data are reported as the median of the percentage of variation for each analyte. Mann Whitney Test was applied to compare the overall median of variations of each stimulation between COVID-19 patients and NO COVID-19-VCs.

To better understand the effect of IL-10 treatment on immune specific response, we evaluated the median variation of each analyte and the overall variation for each group (**Table S2**, **Figure 6**). IL-10 treatment induced a downregulation of the majority of analytes in COVID-19 and in NO COVID-19-VCs in a similar way. After spike stimulation, an overall median decreased percentage of immune factors of 45.4% in COVID-19 and 38.2% in NO COVID-19-VCs was detected. Moreover, for SEB stimulation, an overall median decrease of 38.1% in COVID-19 and 24.9% in NO COVID-19-VCs was observed (**Table S2**). As expected, the median decrease with the SEB was lower than with spike stimulation likely due to the very strong response induced by SEB. Comparing the percentage of median variation of each analyte and the overall median variation showed no significant difference in the majority of cases, neither with spike nor with SEB stimulation (**Table S2**). Similarly, when spike-induced or SEB-induced overall median variations were compared between COVID-19 patients and NO COVID-19-VCs, no significant difference was detected (p=0.395 and p=0.071, respectively) (**Figure 6**).

### IL-10 does not alter cell viability

Then, by flow cytometry, we investigated in 8 NO COVID-19-VCs whether the significant downregulation of the SARS-CoV-2 specific and SEB responses was associated with cell death. SARS-CoV-2 and SEB stimulation did not cause a significant higher cell mortality compared to unstimulated cells; the IL-10 treatment did not affect cell survival of any of the condition tested (**Figure S9**).

### IL-10 modulates the frequency of cytokine producing CD4^+^ and CD8^+^ cells

By flow cytometry, TNF-α and IL-2 producing CD4^+^ T-cells in response to spike stimulation were detected in all vaccinated controls (8 subjects) and IFN-γ producing CD4^+^ T-cells were detected in 7/8 subjects tested.

**Figure 7:**
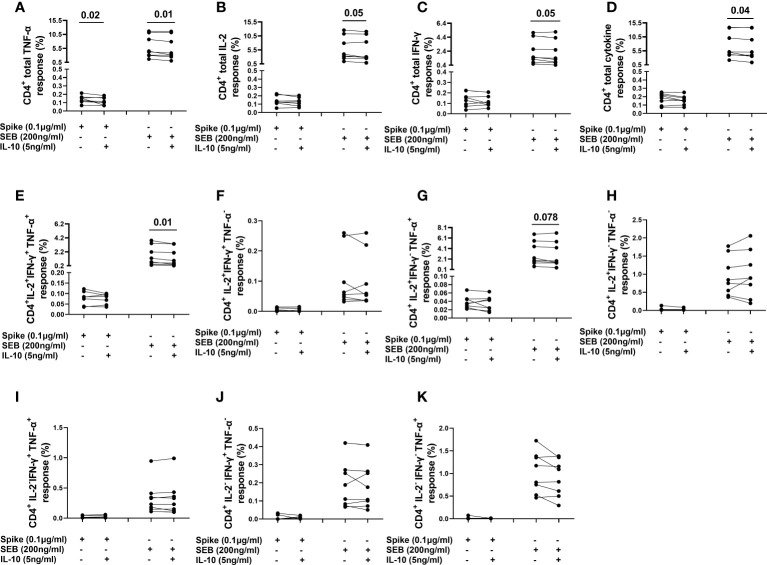
Evaluation of the effect of IL-10 on CD4^+^ T-cells populations in NO COVID-19-Vaccinated Controls of study population “B”, by flow cytometry (n=8). **(A)** percentage of CD4^+^TNF-α^+^ cells; **(B)** percentage of CD4^+^IL-2^+^ cells; **(C)** percentage of CD4^+^IFN-γ^+^cells; **(D)** percentage of total (Th1) cytokine response of CD4^+^ cells; **(E)** percentage of CD4^+^IL-2^+^IFN-γ^+^TNF-α^+^ subset; **(F)** percentage of CD4^+^IL-2^+^IFN-γ^+^TNF-α^-^ subset; **(G)** percentage of CD4^+^IL-2^+^IFN-γ^-^TNF-α^+^ subset; **(H)** percentage of CD4^+^IL-2^+^IFN-γ^-^TNF-α^-^ subset; **(I)** percentage of CD4^+^IL-2^+^IFN-γ^+^TNF-α^-^ subset CD4^+^IL-2^-^IFN-γ^+^TNF-α^+^ subset; **(J)** percentage of CD4^+^IL-2^-^IFN-γ^+^TNF-α^-^ subset; **(K)** percentage of CD4^+^IL-2^-^IFN-γ^-^TNF-α^+^.

The frequency of spike-induced CD4^+^TNF-α^+^ T-cells significantly decreased [0.139 (0.115-0.163) vs 0.107 (0.077-0.163), (p=0.02)] after adding IL-10 (**Figure 7A**). Although not significant, a decreasing trend was also observed in the frequency of IFN-γ^+^ and IL-2 producing CD4^+^ T-cells (**Figures 7B** **, C**). Moreover, IL-10 significantly decreased the frequencies of CD4^+^TNF-α^+^ [4.387 (3.038-10.57) vs 3.521 (2.637-10.370), (p=0.01)], CD4^+^IL-2^+^ [3.717 (2.675-10.44) vs 2.907 (2.55-10.48), (p=0.05)], CD4^+^IFN-γ^+^ [1.507 (0.898-4.123) vs 1.308 (0.782-3.884), (p=0.05)] T-cells, CD4^+^IL-2^+^IFN-γ^+^TNF-α^+^ [1.028 (0.439-03.117) vs 0.775 (0.408-3.021), (p=0.01)] subset, and CD4^+^ total cytokine response [5.08 (4.07-12.84) vs 4.532 (3.562-12.69), (p=0.04)] after SEB stimulation (**Figures 7A** **–E**). However, no significant difference was found neither in the percentages of other subsets (**Figures 7F–K**), nor in CD4+ cells expressing HLA-DR in response to spike or SEB stimulation (**Figures S10A–D**).

**Figure 8:**
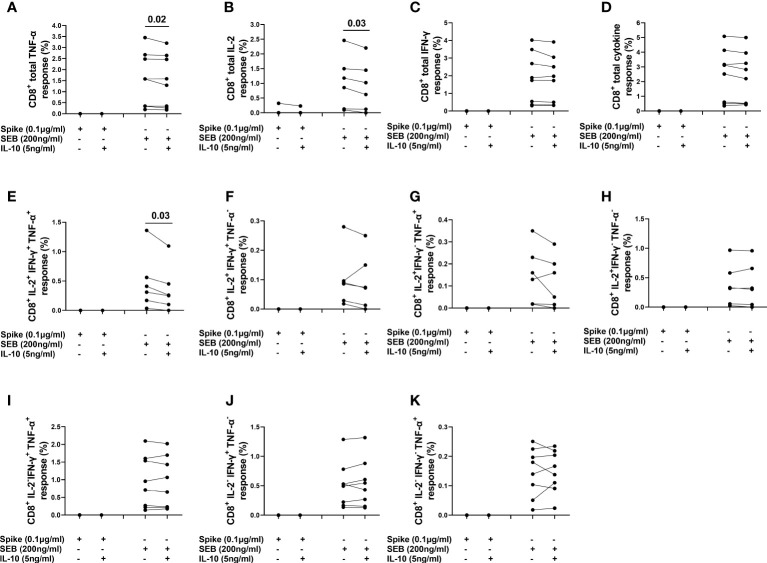
Evaluation of the effect of IL-10 on CD8^+^ T-cells populations in NO COVID-19-Vaccinated Controls of study population “B”, by flow cytometry (n=8). **(A)** Percentage of CD8^+^TNF-α^+^ cells; **(B)** percentage of CD8^+^IL-2^+^ cells; **(C)** percentage of CD8^+^IFN-γ^+^cells; **(D)** percentage of total cytokine response of CD8^+^ cells; **(E)** percentage of CD8^+^IL-2^+^IFN-γ^+^TNF-α^+^ subset; **(F)** percentage of CD8^+^IL-2^+^IFN-γ^+^TNF-α^-^ subset; **(G)** percentage of CD8^+^IL-2^+^IFN-γ^-^TNF-α^+^ subset; **(H)** percentage of CD8^+^IL-2^+^IFN-γ^-^TNF-α^-^ subset; **(I)** percentage of CD8^+^IL-2^+^IFN-γ^+^TNF-α^-^ subset CD8^+^IL-2^-^IFN-γ^+^TNF-α^+^ subset; **(J)** percentage of CD8^+^IL-2^-^IFN-γ^+^TNF-α^-^ subset; **(K)** percentage of CD8^+^IL-2^-^IFN-γ^-^TNF-α^+^.

The spike-induced CD8^+^ T-cells producing IFN-γ and TNF-α (CD8^+^IFN-γ^+^ and CD8^+^TNF-α^+^) T-cells were not detected in any subject and CD8^+^ IL-2^+^ T-cells were detected in only one subject. Differently, the SEB-induced CD8^+^IFN-γ^+^ and CD8^+^TNF-α^+^ T-cells were detected in all subjects and CD8^+^IL-2^+^ T-cells were detected in 6/8 (75%) subjects (**Figures 8A–C**). IL-10 significantly reduced the frequencies of CD8^+^TNF-α^+^ [1.574 (0.345-2.626) vs 1.437 (0.282-2.60), (p=0.02)], CD8^+^IL-2^+^ [1.018 (0.122-1.739) vs 0.823 (0.091-1.637), (p=0.03)] T-cells and also CD8^+^IL-2^+^IFN-γ^+^TNF-α^+^ subset [0.360 (0.136-0.760) vs 0.255 (0.075-0.611), (p=0.03)] after SEB stimulation (**Figures 8A, B, E**). No significant difference was detected in the percentage of other CD8+ subsets (**Figures 8D, F–K**)]. In addition, a significant difference was found in the percentage of CD8^+^TNF-α^+^HLA-DR^+^ [47.2 (38.8-56) vs 43.9 (36.1-53.8), (p=0.05)] and CD8^+^IL-2^+^HLA-DR^+^ [26.7 (5.38-42.1) vs 21.3 (0.0-40.5), (p=0.03)] T-cells in response to SEB stimulation (**Figures S10F, G**).

As expected, no subject showed Natural Killer (NK) cells response to spike stimulation, whereas after SEB stimulation NK cells produced TNF-α, IFN-γ, or IL-2 in few subjects (2/8, 2/8, and 3/8 subjects, respectively).

Il-10 decreased the frequency of these cells; however, the low number of responders did not allow an appropriate statistical analysis (data not shown).

Finally, IL-10 significantly decreased the percentage of SEB-stimulated NK cells expressing HLA-DR [5.69 (2.20-6.47) vs 4.21 (1.84-5.43), (p=0.0078)] (**Figure S10I**).

## Discussion

COVID-19 pandemic is now better controlled in terms of outcome of disease severity by therapies protocols (19–21) and by the vaccine campaign (29); however, although large-scale vaccine administration is available in the majority of countries, identification of new effective therapies is still crucial for those unvaccinated or those that, although vaccinated, get infected and progress to severe disease. Several drugs with immune-modulatory functions are now used for the routine therapy of COVID-19. Some of them, as baricitinib, have been shown to modulate the immune response including the of the SARS-CoV-2- specific T-cell response (21, 30, 31).

In the present study, we showed that IL-10 is significantly increased after 2 weeks from symptoms onset in the plasma of COVID-19 patients likely acting as an internal control cytokine to block the over-expression of several endogenous immune factors. Moreover, we showed for the first time in a whole-blood experimental setting, that exogenous *in vitro* addition of IL-10 decreases SARS-CoV-2-specific response of several immune factors including Th1, Th2, Th17, and chemokines. The IL-10 effect was observed in both, COVID-19 patients and NO COVID-19-VCs.

Interestingly, most of the vaccinated individuals with COVID-19 responding to spike stimulation (5/8) had moderate disease while most of non-responders (5/6) had severe or critical disease (**Table 2**) (one had moderate, 4 had severe, one had critical disease). These results likely reflect the immune impairment of COVID-19 patients with severe to critical symptoms, as previously reported (5).

In this study we confirmed that the spike-specific response in COVID-19 patients is characterized by the predominance of Th1 and low magnitude of Th2 cytokines (23) and we showed ex-vivo, in whole-blood cells of COVID-19 patients, that IL-10 downregulates a broad range of immune factors such as IFN-γ, pro-inflammatory cytokines (IL-1β, IL-6, IL-12, and TNF-α), IL-17, Th2 cytokines (IL-4, IL-5, IL- 13), IL-1RA, growth factors (IL-2, IL-7, IL-15, G-CSF, GM-CSF, and FGF-basic), and chemokines (IL-8, IP- 10, MCP-1, MIP-1α, and MIP-1β). These factors produced by both adaptive and innate immunity cells are known to be increased in COVID-19 patients (32), and associate with COVID-19 severity (12), although recent studies indicate that adaptive immune response is crucial to limit the severity of the disease (33) and for the long-lasting immunity to vaccines (9, 34, 35).

Our results indicate in *ex-vivo* experiments that exogenous addition of IL-10 to whole-blood cells of COVID-19 patients reduces the SARS-CoV-2-specific immune response (36). We showed that IL-10 treatment downregulates the production of several immune factors in a similar way, independently of the COVID-19 status. Moreover, our results indicate that IL-10 acts well on both the SARS-COV-2 specific and the positive control response.

Furthermore, in the present study we observed FGF modulation by IL-10. FGF has shown to be associated with severe disease and Intensive Care Unit (ICU) admission and this modulation may affect the COVID-19 outcomes (37, 38). Consistent with previous studies (1, 12, 38), we also found that IL-5, G-CSF, IP-10, and MCP-1 were associated with severe/critical disease and among them MCP-1 was also associated with mild/modarete disease

The initiation of inflammatory responses is required for an effective immune response against harmful pathogens, however if remains unbalanced, can result in inflammatory disorders, autoimmunity and even cancers (39) or in the case of COVID-19 can lead to dramatic outcome. IL-10 is an important negative regulator of cell-mediated immunity (40). Previous studies have shown that IL-10 can suppress the infection of several viral diseases including skin infection by the poxvirus vaccinia (VV) (41), murine influenza virus infection (42), acute respiratory syncytial virus infection (43), Neurotropic Coronavirus Encephalomyelitis (44), acute influenza virus infection (4). Moreover, IL-10 has a protective role for the lung in viral infections as Influenza (4) and acute respiratory syncytial virus (RSV) infection (43). Dysregulation of IL-10 has shown to be linked with susceptibility to numerous infectious diseases such as Mycobacterium avium Infection (45), Helicobacter hepaticus-induced colitis (46), HIV (47), hepatitis C virus (HCV) infection (48) and autoimmune diseases like inflammatory bowel disease (39, 49). *In vivo* blockade of its action in infected animals resulted in increased pulmonary inflammation and lethal injury (4). These data together suggest that IL-10 may be a promising cytokine to target for treatment of infections and inflammatory diseases (50) *via* decreasing IL-2 secretion by T cells, suppressing activated macrophages and dendritic cells (DC), diminishing the production of cytokines required for effective T helper responses, and reduction of HLA class II expression (40, 50).

We demonstrated that IL-10 treatment reduces the TNF-α, IL-2, and IFN-γ production of CD4^+^ T-cells, CD8^+^ T-cells and NK cells from peripheral blood stimulated with SEB. Similar trend was found in response to spike stimulation, although the difference reached significance only for TNF-α, likely due to the small sample size and the lower magnitude of immune response to spike compared to SEB. In addition, we found that IL-10 significantly decreases HLA-DR expression on the surface of lymphocytes particularly within CD8^+^ T-cells and NK cells confirming the ability of IL-10 to reduce cell activation (40, 50).

It is critical to have a better understanding of how IL-10 is regulated in COVID-19. Our results may improve the knowledge of the complex regulation of cell-mediated cytokine production by IL-10 and confirm a regulatory effect in which IL-10 directly restricts pro-inflammatory cytokine production induced by SARS-CoV-2. However, it should be taken into consideration that any intervention to reduce inflammatory responses may affect negatively on viral clearance (51) and therefore the potent anti-inflammatory drugs need to be provided after the early time points of infection. It is important to note that in other infectious diseases, i.e. tuberculosis, a decreased of M. tuberculosis T-cell specific response has been associated to cured TB patients (52–57). However, for the viral infection control, evidence indicates that the cellular response, mainly Th1-mediated, is essential (35, 58).

To date, the therapeutic administration of recombinant human IL-10 has been studied in clinical trials for different diseases such as rheumatoid arthritis, inflammatory bowel disease, and psoriasis (59, 60). Subcutaneous IL-10 administered daily for 28 days to patients with mild to moderately active Crohn’s disease was found to be safe, well-tolerated, and showed clinical and endoscopic intestinal improvement (61). Pegylated IL-10 (AM0010) also resulted an acceptable safety profile with early evidence of clinical activity in patients with advanced solid tumor malignancies (62).

Few clinical trials have also been conducted for infectious diseases therapy. Nelson et al. demonstrated that IL-10 decreased hepatic inflammation and improved liver histology and function as well as reduced the liver fibrosis in a large proportion of patients with hepatitis C virus (HCV) infection (63). Another report demonstrated that although IL-10 therapy improves various inflammatory parameters and liver fibrosis in patients with chronic HCV infection, in the long term it may lead to increased HCV burden *via* alterations in immunologic viral surveillance (64).

Although IL-10 provides a great opportunity for the treatment of diverse diseases, no therapy indication has been approved so far (60). Despite its anti-inflammatory functions, IL-10 is also highly pleiotropic and elicits diverse and apparently opposing biological effects. Indeed, IL-10 may act as a pro-inflammatory and immunostimulatory molecule under certain conditions. At high dose it may induce systemic immune activation, as evidenced by the production of proinflammatory (IFN-γ) cytokine and T-cells activation and proliferation (14). Thus, administration of lower doses of this cytokine for the therapeutic purposes should be considered. Furthermore, engineered IL-10 agonists may have significant clinical implications for their ability to downregulate IFN-γ production by CD4+ and CD8^+^ T cells and suppress the inflammatory monocyte and macrophage activation (65). Therefore, increasing our understanding of the diverse biology of IL-10 and innovative targeting and delivery strategies, will help us to modulate this cytokine pathways in a disease-specific manner to bring it to clinics (60).

This study has some limitations including the relatively small size of the cohorts (127 subjects) and the monocentric design. However, by an easy to-use whole-blood platform, we showed that the effect of IL-10 was robust on the modulation of specific response to SARS-CoV-2 analyzed on a broad range of immune factors, in both COVID-19 patients and vaccinated individuals. Another limitation, in this study we evaluated by flow cytometry only the IFN-γ, IL-2, and TNF-α (Th1) response; however, with a wider panel of flow cytometry analysis we could have a better understanding of how IL-10 specifically modulates other immune factors besides these cytokines. Moreover, we did not identify the main cell type with IL-10 receptor expression or the main source of IL-10 production.

In conclusion, our study gives important insights into immune modulating effects of IL-10 in COVID-19 on a broad range of immune cells (T and NK cells), inflammatory cytokines, chemokines and growth factors which can provide valuable information regarding the further *in vivo* evaluation of the immunomodulatory effect of IL-10 on inflammatory diseases like COVID-19. Whether these findings translate to clinical influences for COVID-19 therapy remains to be evaluated by randomized, controlled, clinical trials with a large sample size.

## Data availability statement

The raw data supporting the conclusions of this article will be made available by the authors, without undue reservation.

## Ethics statement

The studies involving human participants were reviewed and approved by The Ethical Committee of Lazzaro Spallanzani National Institute of Infectious Diseases (59/2020, 72/2015, 247/2021, 297/2021). The patients/participants provided their written informed consent to participate in this study.

## Author contributions

DG, AA conceived and designed the study. Preliminary experiments were done by AA, DN, VC. Final experiments were performed by SN-F, VV, AS, AMGA, and CF. SN-F, EP, CF, LP, TA and AN analyzed the data and revised the manuscript. FP, EN, DG, GG enrolled patients and GC collected the clinical data. SN-F, DG, and AA drafted the article. All authors critically discussed and interpreted data, and contributed to the article and approved the submitted version.

## Funding

This work was supported by: Funding from Italian Ministry of Health Ricerca Finalizzata COVID-2020-12371849 (DN), 0000395 del 25/05/2021, COVID-2020-12371675, Ricerca Corrente funded COVID-2020-12371849 (DN), 0000395 del 25/05/2021, COVID-2020-12371675 Ricerca Corrente funded by Italian Ministry of Health, and by generous liberal donations funding for COVID-19 research from Esselunga S.p.A, Camera di Commercio, Industria e Artigianato di Roma, Società Numero Blu Servizi S.p.A., Fineco Bank S.p.A, Associazione magistrati della Corte dei conti, and Società Mocerino Frutta Secca s.r.l. (resolutions n°395 of May 25th 2021, n°254 of April 24th 2021 and n°257 of April 14th 2021). The funders were not involved in the study design, collection, analysis, interpretation of data, the writing of this article or the decision to submit it for publication.

## Acknowledgments

The authors gratefully acknowledge the patients that participated to the study.

## Conflict of interest

The authors declare that the research was conducted in the absence of any commercial or financial relationships that could be construed as a potential conflict of interest.

## Publisher’s note

All claims expressed in this article are solely those of the authors and do not necessarily represent those of their affiliated organizations, or those of the publisher, the editors and the reviewers. Any product that may be evaluated in this article, or claim that may be made by its manufacturer, is not guaranteed or endorsed by the publisher.
