# Supplementary material for: Evaluation of the immunomodulatory effects of interleukin-10 on peripheral blood immune cells of COVID-19 patients: Implication for COVID-19 therapy
Source: Front Immunol. 2022 Sep 6;13:984098. doi: 10.3389/fimmu.2022.984098 (PMC9486547; doi:10.3389/fimmu.2022.984098)
Supplement: Supplementary file 1 [file DataSheet_1.docx]

Supplementary Material

- Figure S1
- Figure S2
- Figure S3
- Figure S4
- Figure S5
- Table S1
- Figure S6
- Figure S7
- Figure S8
- Table S2
- Figure S9
- Figure S10


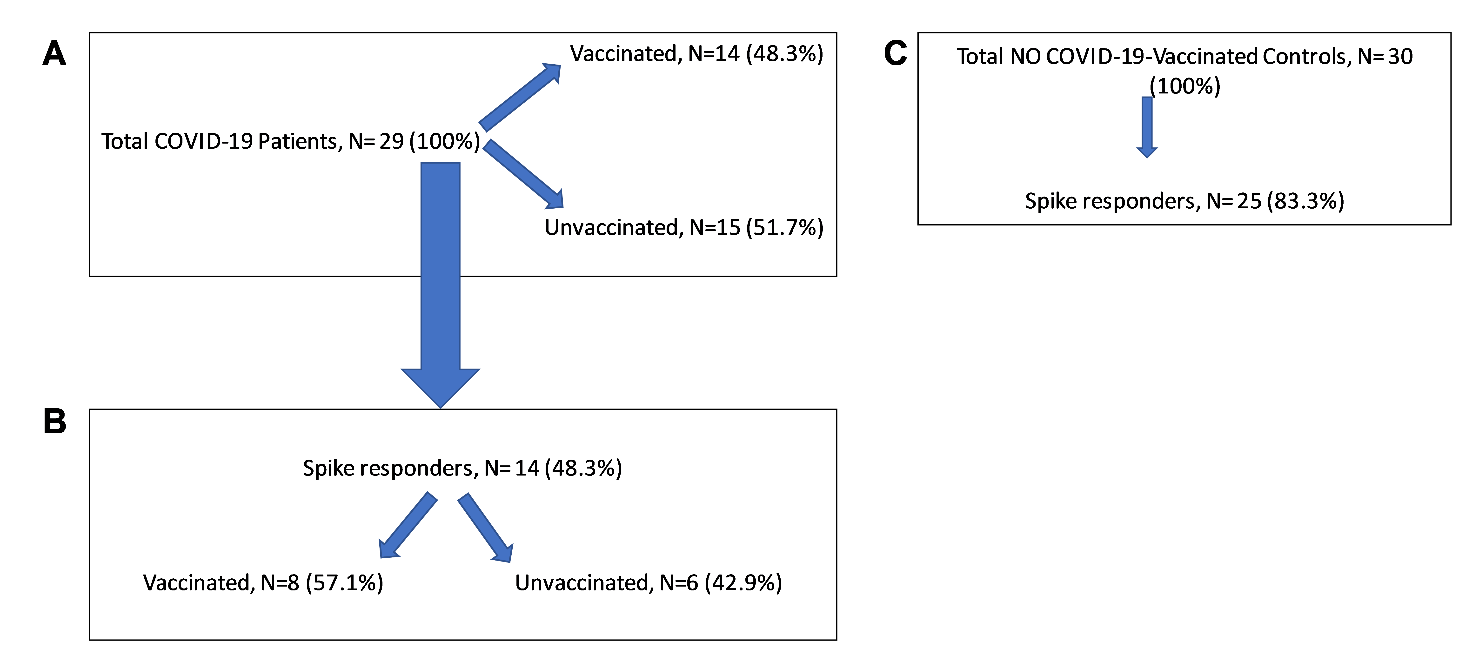


**Figure S1. Clinical characteristics of the enrolled subjects for IL-10 Study** **(study population “B”).** IFN-γ production in response to Spike (Pool S) was considered positive based on a cut-off (0.13 IU/mL) previously defined (24). **A.** Total number of 29 COVID-19 patients including 14 previously vaccinated against COVID-19 (48.3%) and 15 unvaccinated (51.7%) were enrolled. **B.** Within COVID-19 group, 14 (48.3%) patients responded to Spike stimulation by IFN-γ production including 8 (57.1%) vaccinated and 6 (42.9%) unvaccinated. **C.** Total number of 30 controls vaccinated against COVID-19 (NO COVID-19-Vaccinated Controls) were enrolled and among them 25 (83.3%) responded to Spike stimulation.


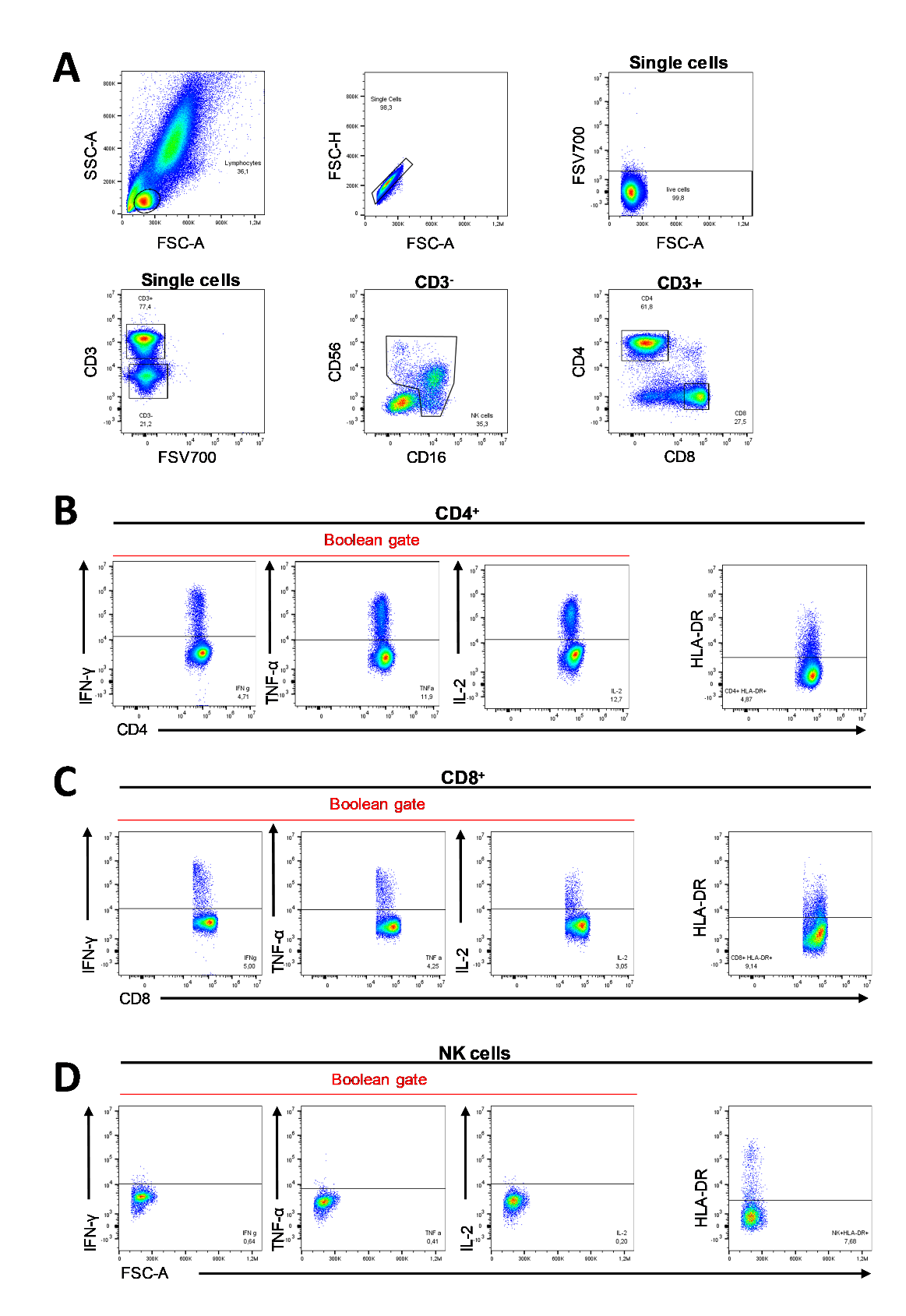


**Figure S2. Flow cytometry gating strategy.** Cell subpopulations were gated as described in the methods section in 8 samples from NO COVID-19-vaccinated controls (NO COVID-19-VCs) according to the expression of surface markers and intracellular cytokine production.


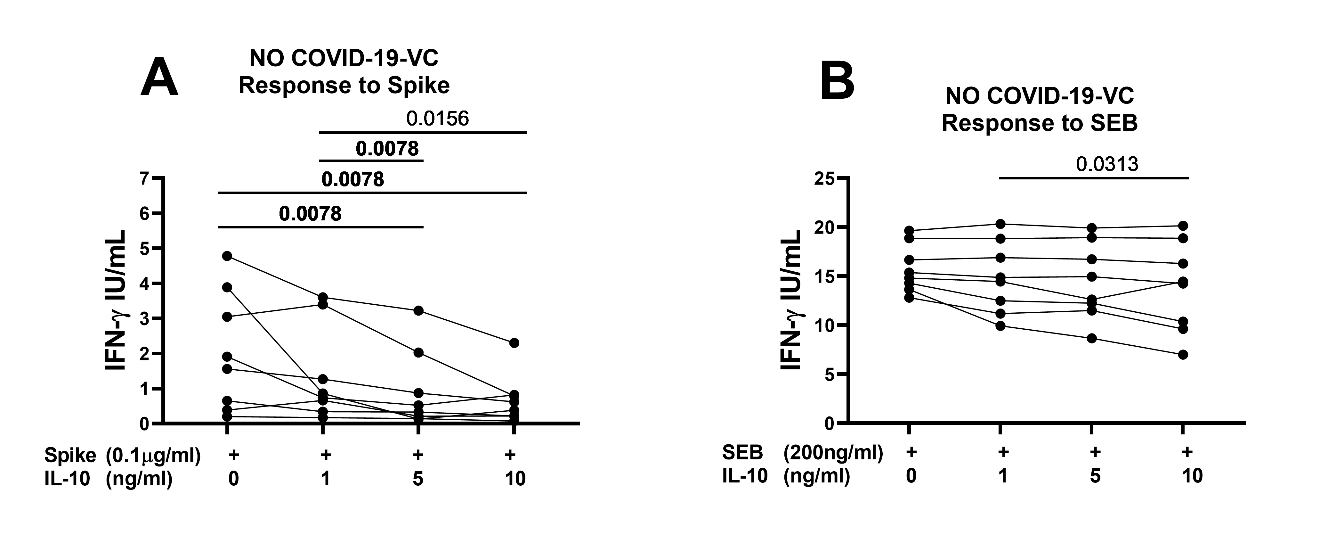


**Figure S3. IL-10 significantly downregulates Spike-induced IFN-γ response in NO COVID-19-Vaccinated Controls (NO COVID-19-VC) of study population “B” (n=8). A.** response to Spike (Pool S) stimulation, **B.** response to SEB stimulation. The downregulation of SEB-induced IFN-γ response, however, did not reach significance. Moreover, no significant difference was detected between IL-10 at 5 ng/ml and at 10 ng/ml neither in response to Spike nor to SEB. After correction for multiple comparisons, p≤ 0.008 was considered as significant.


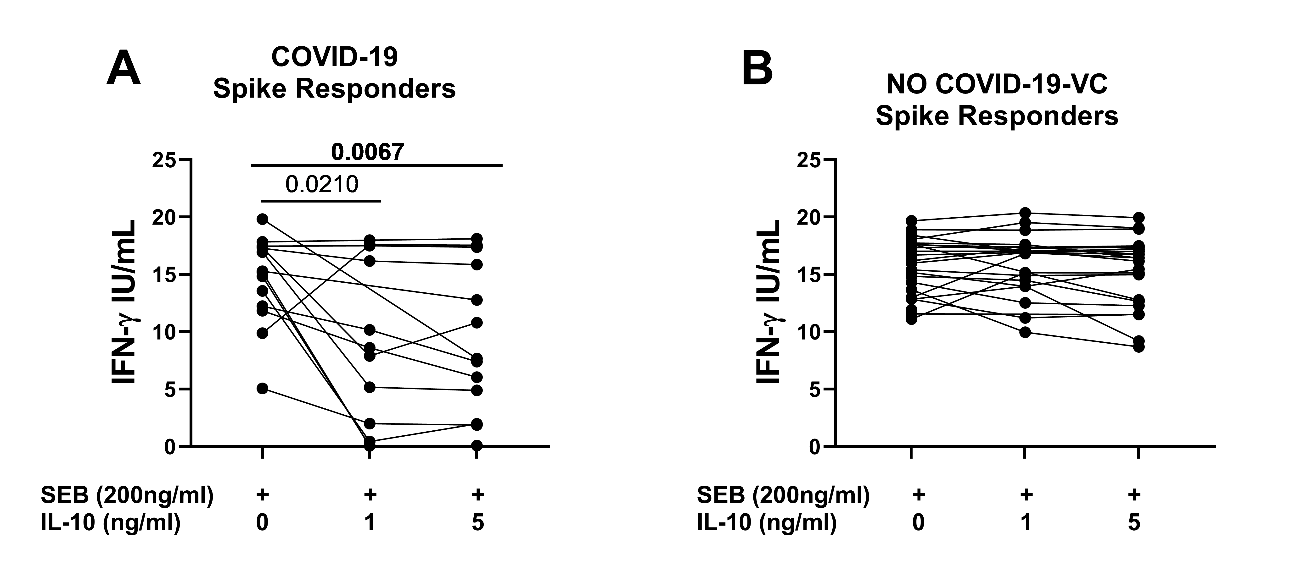


**Figure S4. IL-10 significantly downregulates SEB-induced IFN-γ response in COVI-19 patients of study population “B”. A.** COVID-19 patients, responders to Spike stimulation (n=14), **B.** NO COVID-19-Vaccinated Controls (NO COVID-19-VCs), responders to Spike stimulation (n=25). IFN-γ production in response to Spike (Pool S) was considered positive based on a cut-off (0.13 IU/mL) previously defined (24). After correction for multiple comparisons, p≤ 0.016 was considered as significant.


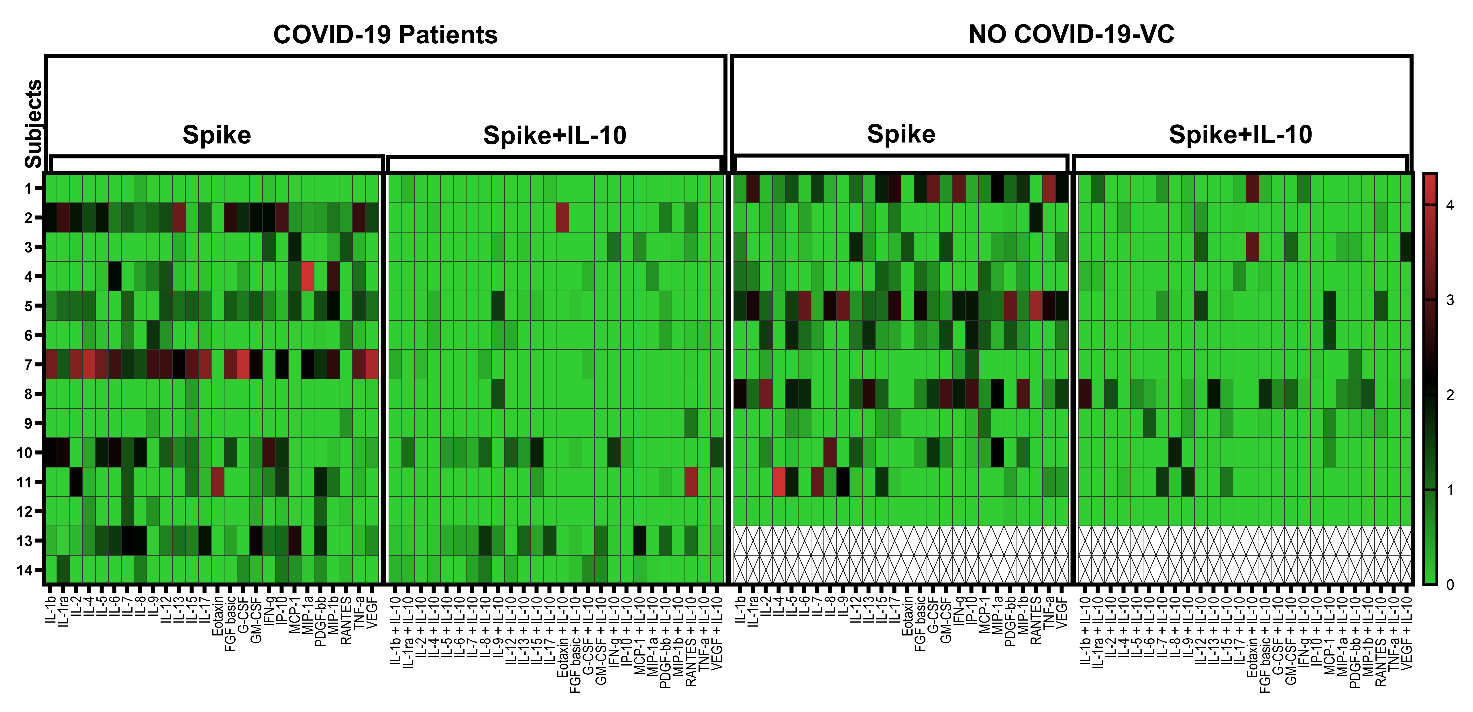


**Figure S5. The effect of IL-10 on SARS-COV-2 specific response in COVID-19 patients (n=14) and NO COVID-19-Vaccinated Controls (NO COVID-19-VC) (n=12)** **of study population “B”, using a heatmap.** Each immune factor value was normalized by subtracting the mean cytokine value calculated for each specific cytokine within each group. Subsequently, this value was divided by the standard deviation calculated for the specific cytokine within each group. Color codes refer to “red” for the highest expression and “green” for the lowest expression levels.

| **Table S1: IL-10 significantly downregulates several SEB-induced cytokines, chemokines, and growth factors in COVID-19 patients and NO COVID-19-VCs of study population “B”, using a 27-plex multiplex assay.** | | | | | | | |
| --- | --- | --- | --- | --- | --- | --- | --- |
|  |  | **COVID-19 Patients** (n=13)^$^ | | | **NO-COVID-19-VC** (n=12) | | |
| **Function** | **Analyte** | **SEB Median (IQR)** | **SEB + IL-10  Median (IQR)** | **P*** | **SEB Median (IQR)** | **SEB + IL-10  Median (IQR)** | **P*** |
| Pro-Inflammatory  cytokine/chemokine | **IL-1β** | 39.44 (17.14-149.8) | 14.84 (6.72-43.36) | 0.0002 | 157.4 (19.09-738.5) | 91.3 (10.6-264.1) | 0.0049 |
|  | **IL-6** | 761.4 (245.8-5591) | 259.9 (69.16-958.9) | 0.0005 | 3444 (736.4-7032) | 1134 (420.1-4709) | 0.0005 |
|  | **IL-8** | 23970 (12486-35529) | 14872 (3525-20291) | 0.0017 | 16472 (11256-65748) | 13948 (7580-50063) | 0.129 |
|  | **IL-12** | 51.44 (34.5-6.16) | 28.8 (16.68-44.74) | 0.0017 | 112.9 (68.85-182.8) | 54.48 (33.63-66.72) | 0.0005 |
|  | **IL-17A** | 122.9 (63.1-224.1) | 53.2 (25.86-135.2) | 0.0081 | 178.2 (115.3-276.2) | 129.5 (70.75-226) | 0.0005 |
|  | **IFN-γ** | 8396 (2105-14084) | 2436 (330.8-8345) | 0.0002 | 9815 (3907-19844) | 5164 (1683-13325) | 0.0005 |
|  | **IP-10** | 14895 (5416-16919) | 7056 (2180-11421) | 0.0005 | 13571 (9794-20267) | 6227 (4319-9216) | 0.0005 |
|  | **MIP-1α** | 2149 (649.5-3770) | 1004 (176.7-1838) | 0.0017 | 2251 (1899-3201) | 2092 (1116-2771) | 0.064 |
|  | **MIP-1β** | 9326 (5264-22246) | 2718 (1775-7757) | 0.0002 | 33932 (27830-34051) | 14731 (10075-29846) | 0.0015 |
|  | **RANTES** | 2498 (420.4-4488) | 1298 (58.32-1487) | 0.021 | 3134 (84.87-7960) | 953.9 (29.25-3590) | 0.0273 |
|  | **TNF-α** | 4484 (868.8-6409) | 458 (140.3-2701) | 0.0002 | 3509 (2527-12365) | 2062 (1079-5911) | 0.0005 |
| Anti-inflammatory  cytokine | **IL-1rα** | 11468 (7208-14446) | 8975 (4515-11711) | 0.01 | 12241 (10419-19239) | 9354 (7108-13306) | 0.0005 |
|  | **IL-4** | 14.96 (11.34-20.64) | 10.72 (4.76-14,32) | 0.0005 | 26.96 (13.43-36.54) | 20.16 (12.28-24.82) | 0.0015 |
|  | **IL-13** | 100.6 (12.62-199.7) | 10.04 (6-131.8) | 0.0398 | 204.8 (114.6-338.4) | 165.9 (91.22-288.1) | 0.0425 |
| Growth factor | **IL-2** | 4957 (2336-8573) | 1586 (236.6-5281) | 0.0002 | 6467 (5408-9436) | 3941 (2164-5435) | 0.0005 |
|  | **IL-5** | 375.9 (248.3-452.5) | 195.4 (126.9-247.1) | 0.0002 | 552.8 (348-838.2) | 344 (242.9-509.1) | 0.0005 |
|  | **IL-7** | 24.48 (16.98-30.2) | 14.28 (9.48-21.46) | 0.0134 | 30.58 (20.76-37.36) | 25.54 (16.31-30.82) | 0.0269 |
|  | **IL-9** | 228.7 (139.6-275.2) | 117.5 (27.82-167.5) | 0.0061 | 292.6 (152.3-385.2) | 256.4 (160.5-307.1) | 0.233 |
|  | **IL-15** | 561.4 (502.1-703.9) | 391.4 (259.3-545.1) | 0.0012 | 633.5 (542.5-931) | 569.5 (468.9-709.5) | 0.0024 |
|  | **FGF-basic** | 155.8 (103.3-196.1) | 83.64 (58.72-136.6) | 0.0002 | 204.1 (141.8-304.4) | 173.1 (118.9-223) | 0.0005 |
|  | **G-CSF** | 8420 (5993-9526) | 5783 (2665-7635) | 0.0061 | 9505 (8887-9971) | 8631 (7479-6938) | 0.0034 |
|  | **GM-CSF** | 114.3 (29.22-243.1) | 45.81 (5.9-92.64) | 0.0002 | 200 (126.6-699.4) | 122.8 (63.75-341.9) | 0.0005 |
|  | **PDGF** | 414.6 (277.8-524) | 172.6 (147.6-261.5) | 0.0005 | 631.5 (378.9-1306) | 430.6 (239.1-683.5) | 0.0005 |
|  | **VEGF** | 423.8 (264.7-488.7) | 338 (160.7-413.7) | 0.0061 | 464.1 (389.4-682.8) | 417.4 (321.7-558.4) | 0.0024 |
| **Footnotes:** COVID-19: CoronaVIrus Disease 19; * Wilcoxon matched-pairs signed rank test; ^$^ Missing values for one COVID-19 patient; NO COVID-19-VC: NO-COVID-19 Vaccinated Control; IQR: Interquartile Range; IL: interleukin; IFN: interferon; IP: IFN-γ inducible Protein; MCP: Monocyte Chemoattractant Protein; MIP: Macrophage Inflammatory Protein; RANTES: Regulated upon Activation, Normal T Cell Expressed and Presumably Secreted; TNF: Tumor Necrosis Factor; FGF: Fibroblast Growth Factor; G-CSF: Granulocyte-Colony Stimulating Factor; GM-CSF: Granulocyte-Macrophage-Colony Stimulating Factor; PDGF: Platelet-Derived Growth Factor; VEGF: Vascular-Endothelial Growth Factor. | | | | | | | |


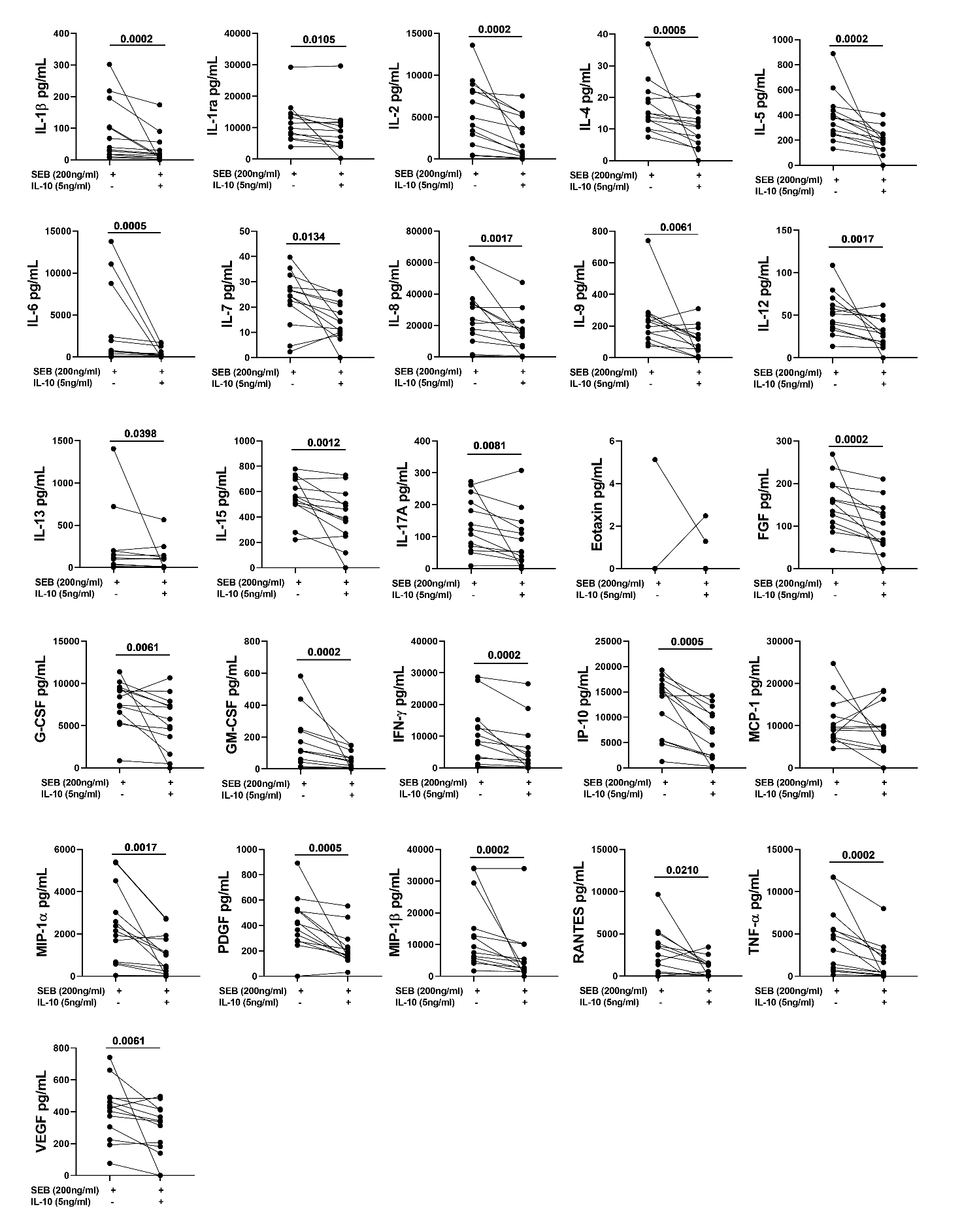


**Figure S6. IL-10 significantly downregulates several SEB-induced cytokines, chemokines, and growth factors in COVID-19 Patients of study population “B”.** Whole-blood was stimulated or not with SEB and then treated or not with IL-10. After overnight stimulation, plasma was collected and used to detect immune factors by a 27-plex multiplex assay (Missing values for one COVID-19 patient, n=13).


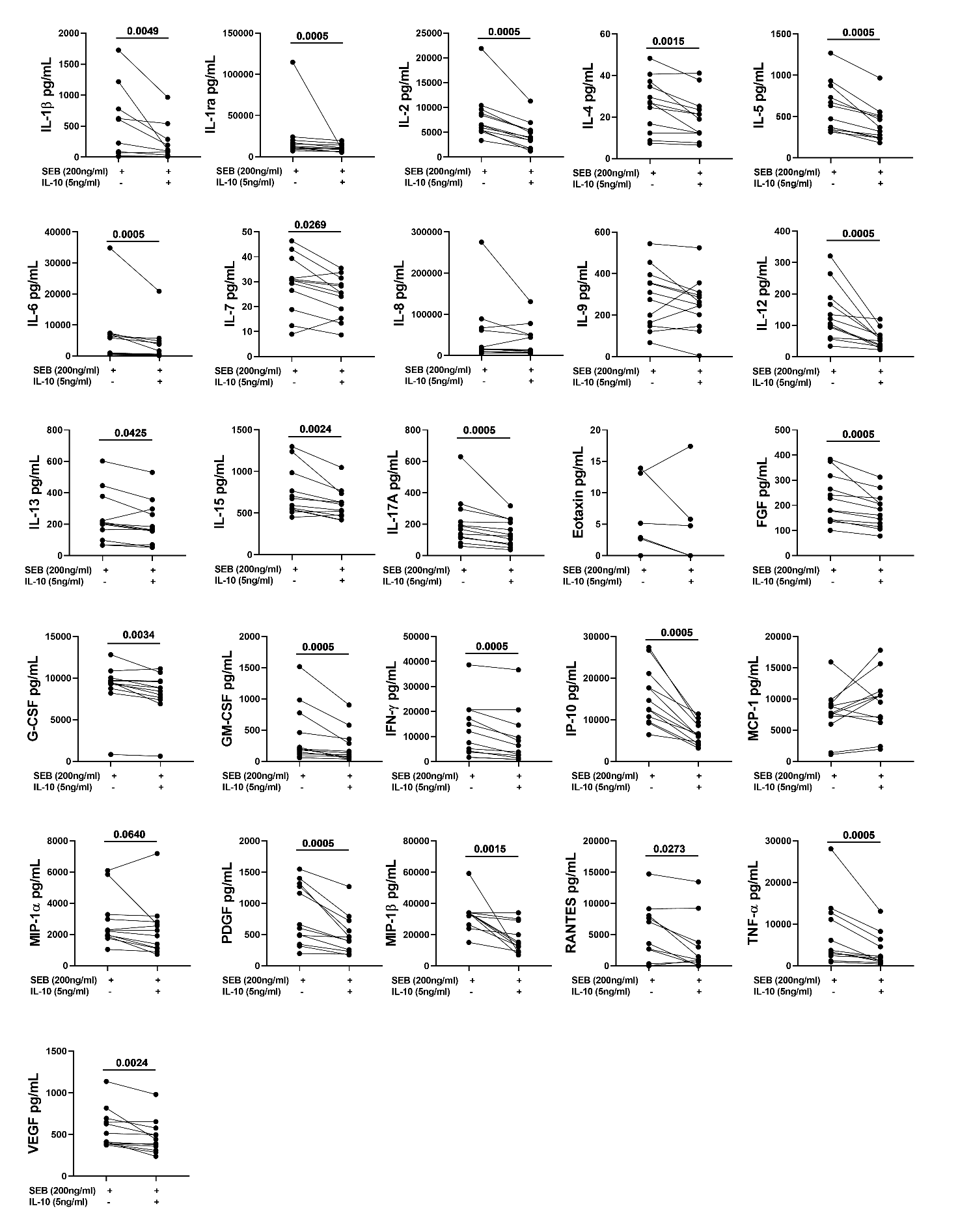


**Figure S7. IL-10 significantly downregulates several SEB-induced cytokines, chemokines, and growth factors in NO COVID-19-Vaccinated Controls (NO COVID-19-VC) of study population “B”.** Whole-blood was stimulated or not with SEB and then treated or not with IL-10. After overnight stimulation, plasma was collected and used to detect immune factors by a 27-plex multiplex assay (n=12).


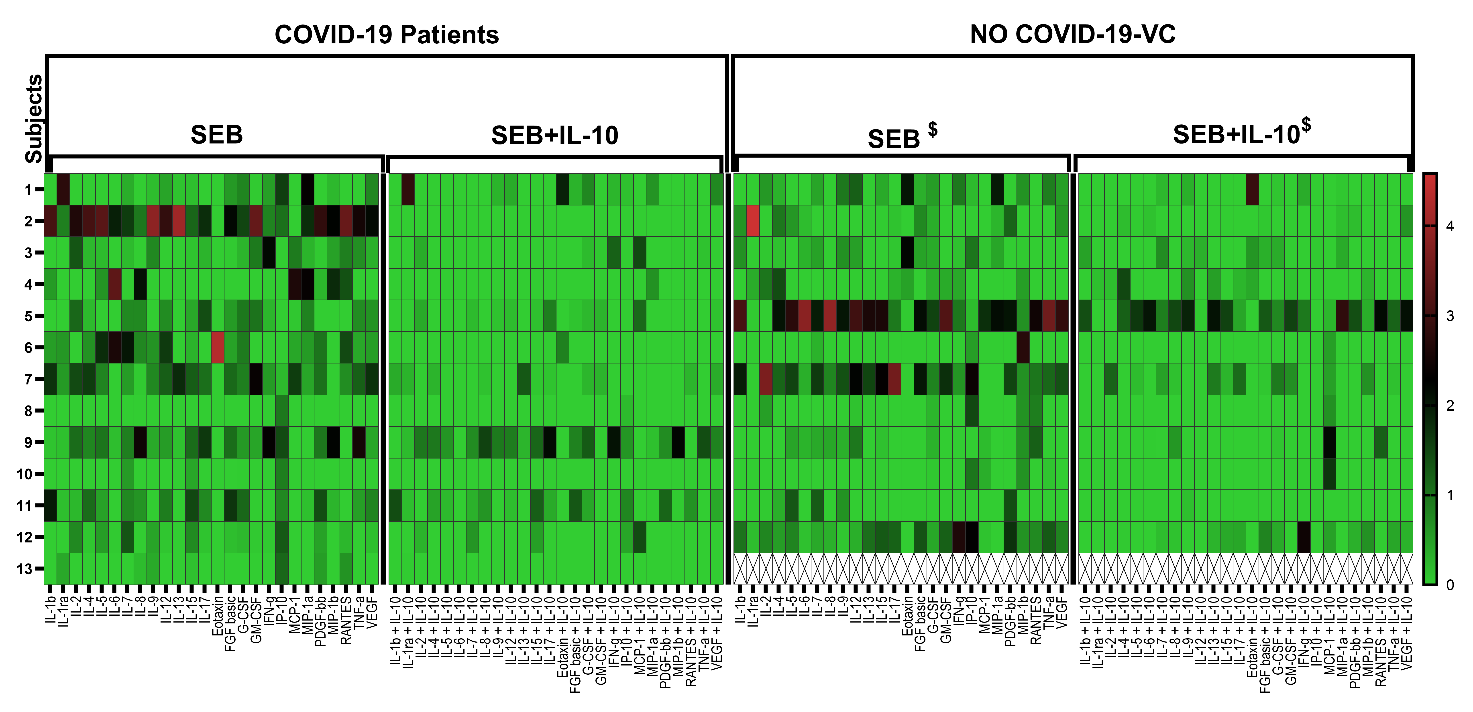


**Figure S8. The effect of IL-10 on SEB-induced response in COVID-19 patients (n=13) and NO COVID-19-Vaccinated Controls (NO COVID-19-VC) (n=12) of study population “B”, using a heatmap.** Each immune factor value was normalized by subtracting the mean cytokine value calculated for each specific cytokine within each group. Subsequently, this value was divided by the standard deviation calculated for the specific cytokine within each group. Color codes refer to “red” for the highest expression and “green” for the lowest expression levels. ^$^ Missing values for one COVID-19 patient.

| **Table S2: IL-10 treatment decreases cytokines, chemokines and growth factors in COVID-19 patients and NO COVID-19-VC subjects of study population “B”.** | | | | | | | | | | | |
| --- | --- | --- | --- | --- | --- | --- | --- | --- | --- | --- | --- |
| **COVID-19** | | | | | | **NO COVID-19-VC** | | | | | |
| **Analyte** | **Spike** | | **SEB** | | **Spike vs SEB** | **Spike** | | **SEB** | | **Spike vs SEB** |  |
|  | **Relative variation after IL-10 treatment (Median %)** | **Relative variation vs the overall relative variation *p**** | **Relative variation after IL-10 treatment (Median %)** | **Relative variation vs the overall relative variation *p**** | **Comparison of the relative variation (%) after IL-10 treatment *p***** | **Relative variation after IL-10 treatment (Median %)** | **Relative variation vs the overall relative variation *p**** | **Relative variation after IL-10 treatment (Median %)** | **Relative variation vs the overall relative variation *p**** | **Comparison of the relative variation (%) after IL-10 treatment *p***** |  |
| **IL-1β** | -29.8 | 0.168 | -39.6 | 0.244 | 0.345 | -45.7 | 0.470 | -50.5 | 0.077 | 0.938 |  |
| **IL-6** | -72.9 | 0.153 | -64.6 | 0.033 | 0.507 | -61.7 | 0.078 | -39.4 | 0.012 | 0.308 |  |
| **IL-8** | -47.2 | 0.903 | -47.4 | 0.787 | 0.221 | -34.8 | 0.470 | -20.3 | 0.677 | 0.182 |  |
| **IL-12** | -20.2 | 0.318 | -28.2 | 0.636 | 0.917 | -59.8 | 0.470 | -61.0 | 0.005 | 0.875 |  |
| **IL-17A** | -55.4 | 0.357 | -25.2 | 0.497 | 0.279 | -35.1 | 0.477 | -31.4 | 0.380 | 1.000 |  |
| **IFN-γ** | -40.2 | 0.346 | -57.7 | 0.127 | 0.507 | -42.0 | 0.204 | -41.0 | 0.052 | 0.638 |  |
| **IP-10** | -71.4 | 0.011 | -48.8 | 0.068 | 0.019 | -81.3 | 0.001 | -59.6 | 0.001 | 0.004 |  |
| **MCP-1** | -22.2 | 0.079 | -4.6 | 0.147 | 0.221 | -12.5 | 0.042 | 43.3 | 0.002 | 0.023 |  |
| **MIP-1α** | -47.9 | 0.583 | -49.7 | 0.110 | 0.311 | -55.9 | 0.516 | -13.9 | 0.470 | 0.028 |  |
| **MIP-1β** | -64.8 | 0.268 | -33.7 | 0.455 | 0.221 | -64.6 | 0.204 | -44.8 | 0.129 | 0.308 |  |
| **RANTES** | 0.0 | 0.010 | -58.5 | 0.497 | 0.142 | 0.0 | 0.002 | -27.4 | 0.835 | 0.365 |  |
| **TNF-α** | -62.7 | 0.296 | -56.0 | 0.002 | 0.753 | -40.1 | 0.977 | -51.8 | 0.002 | 0.583 |  |
| **IL-1RA** | -30.1 | 0.058 | -13.2 | 0.048 | 0.861 | -40.7 | 0.850 | -20.6 | 0.910 | 0.388 |  |
| **IL-4** | -42.5 | 0.843 | -27.5 | 0.636 | 0.807 | -19.2 | 0.834 | -20.3 | 0.266 | 0.308 |  |
| **IL-13** | -21.7 | 0.572 | -25.8 | 0.455 | 0.861 | -37.2 | 0.100 | -20.7 | 0.064 | 0.023 |  |
| **IL-2** | -65.3 | 0.004 | -66.2 | 0.057 | 0.279 | -56.4 | 0.001 | -44.5 | 0.001 | 0.041 |  |
| **IL-5** | -52.7 | 0.296 | -41.4 | 0.685 | 0.101 | -51.9 | 0.339 | -32.5 | 0.064 | 0.158 |  |
| **IL-7** | -59.8 | 0.261 | -21.4 | 0.455 | 0.279 | 1.3 | 0.021 | -20.8 | 0.204 | 0.182 |  |
| **IL-9** | -24.2 | 0.326 | -52.6 | 0.414 | 0.650 | 1.4 | 0.009 | -18.0 | 0.110 | 0.012 |  |
| **IL-15** | -37.8 | 0.730 | -21.6 | 0.127 | 0.650 | -28.3 | 0.339 | -17.4 | 0.016 | 0.117 |  |
| **FGF-basic** | -27.6 | 0.241 | -24.0 | 0.216 | 0.553 | -27.4 | 0.034 | -18.2 | 0.034 | 0.754 |  |
| **G-CSF** | -40.9 | 0.583 | -22.4 | 0.191 | 0.422 | -58.7 | 0.569 | -10.8 | 0.002 | 0.010 |  |
| **GM-CSF** | -51.2 | 0.937 | -59.8 | <0.001 | 0.133 | -35.6 | 0.470 | -38.8 | 0.151 | 0.388 |  |
| **PDGF-BB** | -49.6 | 0.986 | -48.8 | 0.735 | 0.345 | -69.5 | 0.835 | -38.9 | 0.092 | 0.638 |  |
| **VEGF** | -65.8 | 0.855 | -19.0 | 0.244 | 0.650 | -24.8 | 0.424 | -15.2 | 0.092 | 0.754 |  |
| **Eotaxin** | 0.0 | 0.008 | 0.0 | 0.001 | 0.875 | 0.0 | 0.001 | 0.0 | 0.635 | 0.063 |  |
| **Overall** | **-45.4** | **-** | **-38.1** | **-** | **0.126** | **-38.2** | **-** | **-24.9** | **-** | **0.025** |  |
| **Footnotes:** *One sample Wilcoxon signed-rank test. ** Wilcoxon matched-pairs signed-rank; IL: interleukin; IFN: interferon; IP: IFN-γ inducible Protein; MCP: Monocyte Chemoattractant Protein; MIP: Macrophage Inflammatory Protein; RANTES: Regulated upon Activation, Normal T Cell Expressed and Presumably Secreted; TNF: Tumor Necrosis Factor; FGF: Fibroblast Growth Factor; G-CSF: Granulocyte-Colony Stimulating Factor; GM-CSF: Granulocyte-Macrophage-Colony Stimulating Factor; PDGF: Platelet-Derived Growth Factor; VEGF: Vascular-Endothelial Growth Factor. | | | | | | | | | | | |


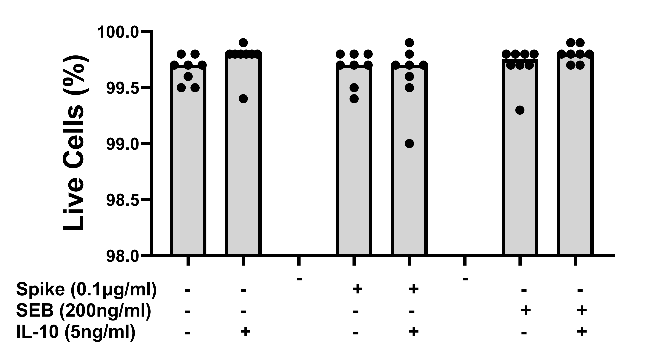


**Figure S9. IL-10 does not modify cell viability of PBMCs.** IL-10 at 5 ng/ml does not affect cell survival of any condition tested.


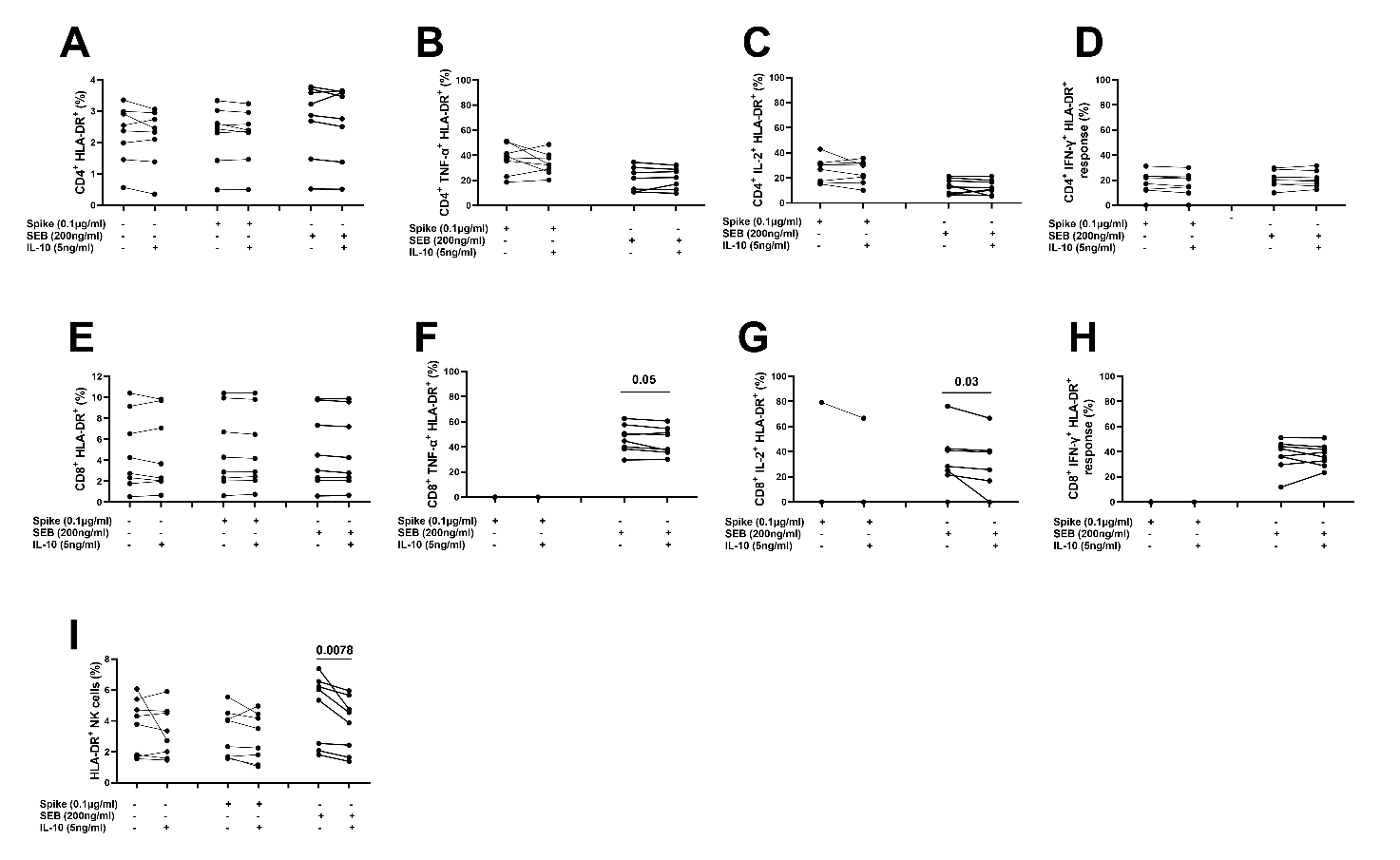


**Figure S10. The effect of IL-10 at 5 ng/ml on HLA-DR expression.** **A.** percentage of total CD4^+^HLA-DR^+^ cells; **B.** percentage of CD4^+^TNF-α^+^HLA-DR^+^ cells ; **C.** percentage of CD4^+^IL-2^+^HLA-DR^+^ cells; **D.** percentage of CD4^+^IFN-γ^+^HLA-DR^+^ cells; **E.** percentage of total CD8^+^HLA-DR^+^ cells; **F.** percentage of CD8^+^TNF-α^+^HLA-DR^+^ cells ; **G.** percentage of CD8^+^IL-2^+^HLA-DR^+^ cells; **H.** percentage of CD8^+^IFN-γ^+^ HLA-DR^+^ cells; **I.** percentage of total NK cells expressing HLA-DR (n=8).
